# Supplementary material for: Fabrication of cytotoxic mirror image nanopores
Source: Nat Commun. 2025 Oct 2;16:8666. doi: 10.1038/s41467-025-64025-6 (PMC12491518; doi:10.1038/s41467-025-64025-6)
Supplement: Supplementary file 1 — Supplementary Information [file 41467_2025_64025_MOESM1_ESM.pdf]

# Supplementary Information

# Fabrication of Cytotoxic Mirror Image Nanopores

Neilah Firzan CA[1,2†], Kalyanashis Jana[3†], Sreelakshmi Radhakrishnan[4,5†], Rifat Aara[6†], Mubeena S[6], Radhika Nair[6], Harsha Bajaj[4,5], Ulrich Kleinekathöfer[3], and Kozhinjampara R Mahendran[1]*

[†]Contributed equally to this work

[1]Membrane Biology Laboratory, Transdisciplinary Research Program, Rajiv Gandhi Centre for Biotechnology, Thiruvananthapuram 695014, India.

[2]Manipal Academy of Higher Education, Manipal, Karnataka, India-576104

[3]School of Science, Constructor University, 28759 Bremen, Germany

[4]Microbial Processes and Technology Division, CSIR- National Institute for Interdisciplinary Science and Technology (NIIST), Thiruvananthapuram 695019, India

[5]Academy of Scientific and Innovative Research (AcSIR), Ghaziabad 201002, India

[6]Centre for Human Genetics, Bengaluru, Karnataka, 560100, India

*To whom correspondence should be addressed

*e-mail: mahendran@rgcb.res.in

## Contents

**Supplementary Information Materials** 4

**Supplementary Information Text** 4

a) Peptide synthesis and analytical size exclusion chromatograpgy 4

b) Ion selectivity measurements through DpPorA and DpPorA DE pores 5

c) Proteinase K enzyme activity on DpPorA and DpPorA DE pores 6

d) Pore design 6

e) Applied electric field MD simulation 7

f) Ramachandran plot of the pores 7

g) Preparation of DDM and Peptides for testing on cancer cells 8

h) Cell membrane integrity analysis and statistics 8

**Supplementary Figures** 9

Figure 1. HPLC traces, mass spectra and CD spectra for DpPorA peptides 9

Figure 2. Single-channel characterization of DpPorA 10

Figure 3. Interaction of DpPorA with E9 peptides 11

Figure 4. Single-channel characterization of DpPorA DE. 12

Figure 5. Interaction of DpPorA DE without and with analytes. 13

Figure 6. Electrical recordings of DpPorA DE without and with analytes. 15

Figure 7. Single-channel characterization of DpPorA DE in different salt conditions and DDM concentrations. 16

Figure 8. Ramachandran plots of the pores. 17

Figure 9. Protein backbone-backbone RMSD values relative to the equilibrated pore structures of LpPorA and DpPorA. 18

Figure 10. Protein backbone-backbone RMSD values relative to the equilibrated pore structures LpPorA DE and DpPorA DE. 19

Figure 11. Structures and electrostatic potentials of LpPorA and DpPorA 20

Figure 12. Structures and electrostatic potentials of LpPorA DE and DpPorA DE. 21

Figure 13. Overlapped structure view of L and D pores 22

Figure 14. The HOLE surface-radius profile along with the designed pore. 23

Figure 15. Electrostatic potential maps of LpPorA and LpPorA DE 24

Figure 16. Translocation of E9 and PEG 200-E9 through DpPorA 25

Figure 17. Transport across DpPorA in Enantiomeric and Cis DOPC giant unilamellar vesicle systems. 26

Figure 18. Effect of DDM and pPorA peptides on MDA-MB-231 cells. 28
Figure 19. Effect of DpPorA DE and LpPorA DE peptides on MDA-MB 231 and MCF 10A cells 30
Figure 20. Fluorescence microscopy images showing two cell membrane integrity phenotypes observed in the MDA-MB-231 cells 31
Figure 21. Single-channel properties of 5-FAM-DpPorA DE. 32
Figure 22. Fluorescence study of 5-FAM-DpPorA DE. 33
**Supplementary Table** 34
Table 1: Average computed conductance values (GMD) for L and D pores 34
Table 2. Concentrations of peptide tested and corresponding DDM concentration 34
Table 3. Effect of peptides on the viability of MDA-MB-231 cells 35
Table 4. Intact and Disrupted phenotypes of cell membrane 36
**References** 37

**Supplementary Information Materials:**

The following materials were used for the study: 1,2-diphytanoyl-*sn*-glycero-3-phosphocholine (DPhPC, Avanti Polar Lipids), pentane (Sigma-Aldrich Merck), hexadecane (Sigma-Aldrich Merck), n-dodecyl β-D-maltoside (DDM, Sigma-Aldrich Merck), potassium chloride (Sigma-Aldrich Merck), 4-(2-hydroxyethyl)-1-piperazineethanesulfonic acid (HEPES, Sigma-Aldrich Merck), 2-propanol (Sigma-Aldrich Merck), methanol (Sigma-Aldrich Merck), cyclic hexasaccharide sulfate ($s_6\alpha$CD, AraChem Cyclodextrin-Shop), cyclic octasaccharide sulfate ($s_8\gamma$CD, AraChem Cyclodextrin-Shop), proteinase K (Sigma-Aldrich Merck), phenylmethylsulfonyl fluoride (PMSF, Sigma-Aldrich Merck), 2x Laemmli sample buffer (Bio-Rad), Any kD™ Mini-PROTEAN® TGX™ precast gel (Bio-Rad), Precision Plus Protein™ Dual Color Standards (Bio-Rad), ATTO-550 DOPE, Mowiol 28-99 (MW 145,000 Da fully hydrolyzed polyvinyl alcohol PVA). Alexa Fluor 350 Hydrazide: M.W-349 Da, Alexa Fluor 555 Hydrazide: M.W-1150 Da, ATTO 488 Dextran: M.W-3000 Da (Thermo-Fischer Invitrogen), Cis-DOPC and Ent-DOPC (Avanti polar lipids), MDA-MB-231, MCF10A, DMEM (Gibco, 11965-092), FBS (Thermo Fisher Scientific, 10270-106), Penicillin (100 Units/mL) /Streptomycin (100 µg/mL) (Thermo Fisher Scientific, 15070-063), DMEM/F12 (Invitrogen, 11330-032), Horse serum (Invitrogen,16050-122), EGF (PeproTech, AF-100-15-1mg), Hydrocortisone: (Sigma-Aldrich Merck, H-0888), Cholera Toxin: (Sigma-Aldrich Merck, C-8052), Insulin (Sigma-Aldrich Merck, I-1882), MTT (3-(4,5-dimethylthiazol-2-yl)-2,5-diphenyltetrazolium bromide, M6494, Thermo Fischer Scientific), DMSO (D5879, Sigma-Aldrich Merck), CellMask Deep Red (Thermo Fisher Scientific), ProLong™ Gold Antifade Mountant (Invitrogen, P10144), All DpPorA peptides, 5-carboxyfluorescein (5-FAM) tagged DpPorA DE peptides, nonaglutamic acid (E9), PEGylated E9 and PEGylated R9 were purchased from GaloreTx Pharmaceuticals Private Limited, Bangalore, India, at >95% purity (HPLC) as lyophilized powders. The alpha-synuclein protein expressed in *E. coli* is purified for single-channel electrical recordings.

**Supplementary Information Text:**

**a) Peptide synthesis and analytical size exclusion chromatograpgy:**

The linear peptide was synthesized using solid-phase peptide synthesis (SPPS) on 2-chlorotrityl chloride (CTC) resin (loading: 0.7 mmol/g). The first Fmoc-protected amino acid was attached to the resin using Fmoc amino acid/DIPEA in dry dichloromethane (DCM). After coupling, the resin was washed sequentially with DCM, DMF, and again with DCM. Following

the initial amino acid attachment, unreacted CTC resin sites were capped using a capping solution composed of 2% DIPEA in dry DCM and methanol (1:1). The Fmoc group of the attached amino acid was deprotected using 20% (v/v) piperidine in DMF. The resin was washed six times each with DMF, DCM, and DMF. A positive ninhydrin test confirmed the complete removal of the Fmoc group. A pre-activated solution of the next Fmoc-protected amino acid, HOBt, and DIC in dry DMF was added to the deprotected resin, and the mixture was shaken at room temperature for 3 hours. The resin was again washed with DMF, DCM, and DMF. A negative ninhydrin test confirmed completion of the coupling reaction. Subsequent Fmoc-protected amino acids were coupled similarly using iterative deprotection and coupling cycles. After the Fmoc-protected peptide synthesis, the Fmoc from the peptidyl resin was removed using 20% piperidine in DMF, and the resin was thoroughly washed with DMF, DCM, and DMF. 5-carboxyfluorescein (5-FAM) was then coupled to the N-terminus using 3 equivalents each of 5-FAM acid, HOBt and DIC in dry DMF. The mixture was shaken for 3 hours at room temperature. A negative ninhydrin test confirmed reaction completion. The resin was then washed with methanol and diethyl ether and dried. Cleavage and deprotection of the peptide were performed using a cleavage cocktail consisting of 95% trifluoroacetic acid (TFA), 2.5% triisopropylsilane (TIPS), and 2.5% water at room temperature. Excess TFA and solvents were evaporated under a nitrogen stream to a small volume. The residue was cooled to 0 °C, and anhydrous diethyl ether was added to precipitate the peptide. The precipitated peptide was collected by centrifugation. The crude peptide was purified using preparative HPLC to obtain the desired product with a purity of 96.3%. Analytical HPLC and mass spectrometry confirmed peptide purity and integrity.

We examined the solubility and folding of DpPorA peptides by analytical size exclusion chromatography (SEC) using different DDM concentrations (0.008% and 0.1%). SEC was performed on a Superdex 200 Increase 10/300 GL column. Peptides solubilized in 0.1% DDM produced a sharp peak in SEC corresponding to peptide folding. Peptides in 0.008% DDM, closer to the critical micelle concentration, showed a small peak in SEC, indicating that a portion of the peptides remained folded. Further, SDS-PADE analysis of the SEC fractions collected between 11 mL and 16 mL confirmed the monomeric peptides (~4 kDa).

**b) Ion selectivity measurements through DpPorA and DpPorA DE pores**

The ion selectivity measurements were performed by establishing a KCl concentration gradient across the bilayer chamber (0.15 M KCl, cis and 1 M KCl, trans). The potential difference was

applied through Ag/AgCl electrodes with agarose salt bridges. The pore formation in the membrane resulted in a current at 0 mV. The 'reverse potential' ($V_m$) was calculated using the Goldman-Hodgkin-Katz equation[1].

$$V_m = \frac{RT}{F} \ln\left(\frac{P_{K^+}[K^+]^{cis} + P_{Cl^-}[Cl^-]^{trans}}{P_{K^+}[K^+]^{trans} + P_{Cl^-}[Cl^-]^{cis}}\right)$$

In this equation, *R* is the universal gas constant (8.314 $J.K^{-1}.mol^{-1}$), *T* is the temperature in Kelvin (K = °C + 273.15), *F* is Faraday's constant (96485 $C.mol^{-1}$), $P_K^+$ is the membrane permeability for $K^+$, $P_{Cl}^-$ is the relative membrane permeability for $Cl^-$, $[K^+]^{cis}$ is the concentration of $K^+$ in the cis side, $[K^+]^{trans}$ is the concentration of $K^+$ in the trans side, $[Cl^-]^{cis}$ is the concentration of $Cl^-$ in the cis side and $[Cl^-]^{trans}$ is the concentration of $Cl^-$ in the trans side. The reverse potential for DpPorA and DpPorA DE was calculated to be +21 mV and +40 mV, respectively.

**c) Proteinase K enzyme activity on DpPorA and DpPorA DE pores**

DpPorA peptides were prepared in 10 mM phosphate buffer with 0.1% DDM (pH 7.4). A final concentration of 0.5 mg/ml of freshly prepared proteinase K solution was added to the samples. The reaction mixtures were heated at 63°C for 30 min. The proteolysis was terminated by adding 2 mM PMSF (phenylmethyl sulfonyl fluoride).[2] The reaction products were loaded on a Mini-PROTEAN® TGX™ precast gel (Bio-Rad) with Protein Standard Marker (Bio-Rad) followed by SDS-PAGE.

**d) Pore design**

Initially, LpPorA and LpPorA DE were designed from their respective sequences using the CCBuilder web server.[3] The octameric structures of the LpPorA and LpPorA DE were constructed from the sequence by adjusting three key parameters: the pitch angle, the radius of the assembly, and the interface angle. Specifically, the LpPorA was designed with a pitch angle of 155 degrees, a radius of 14 Å, and an interface angle of 265 degrees. Similarly, the LpPorA DE was crafted with a pitch angle of 100 degrees, a radius of 14 Å, and an interface angle of 265 degrees. The final pore diameters for LpPorA and LpPorA DE were 14 Å. Subsequently, DpPorA and DpPorA DE were modeled using the BIOVIA Discovery Studio software[4], which facilitated the conversion of the L-isomeric structure to the D-isomeric structure by altering the

handedness of all chiral centers. These initial structures were then subjected to unbiased MD simulation.

**e) Applied electric field MD simulations**

Applied electric field MD simulations were utilized using an external homogeneous electric field E perpendicular to the membrane plane to determine the conductance of KCl through both the wild and mutated LpPorA and DpPorA pores.[5] The corresponding membrane potential V is determined by the product of the applied electric field E and the length $L_z$ of the simulation box in the direction of the applied field, i.e., $V = E * Lz$. The ionic currents are then extracted.[6] The applied field simulations were performed with 1 M KCl and run three times, each time for 100 ns for each pPorA and pPorA DE system. These simulations were conducted at +0.2 V, +0.5 V, -0.2 V, and -0.5 V.

**f) Ramachandran plot of the pores**

Initially, the converted D-pores precisely mirror the structural characteristics of their L-pore counterparts. Subsequently, we analyzed sidechain reorientations resulting from the L-to-D-pore conversions and their impact on stability and ion conductivity. Structural changes induced by the chiral amino acids of the model pore were thoroughly assessed using the PROCHECK webserver.[7]

Our analysis of the Ramachandran plots is based on the final structures obtained from the 500 ns unbiased simulations. For the modeled LpPorA, 97.7% of amino acids were situated in the most allowed region, with an additional 2.3% falling within the allowed region. Notably, no amino acid residues were found in the disallowed region **(Supplementary Fig. 8)**. In the case of DpPorA, 41.4% of amino acids were positioned in the most allowed region, while 50.8% were within the allowed region.[8] Moreover, 5.5% resided in the additionally allowed region, with only 2.3% of amino acid residues in the disallowed region. It is noteworthy that all amino acid residues occupied the left-handed helix region of the Ramachandran plot, except for two D-alanine residues residing there and one D-threonine residue, due to its two chiral centers, which were all three found in the disallowed region.

Moving on to the LpPorA DE and DpPorA DE pores, our analysis revealed that most, i.e., 97.7% of the amino acid residues, reside in the most allowed region, with 1.6% found in the allowed region. At the same time, the Ramachandran plot analysis of DpPorA DE demonstrated that 42.2%, 49.2%, and 6.2% of residues were situated in the most allowed, allowed, and additionally allowed regions, respectively. Notably, 2.3% were in the disallowed region, with

three D-alanine and one D-threonine residues. We note in passing that a few crystal structures of D-amino acid containing proteins, such as 3LQS and 1DAA, also featured 0.8% and 1.0% amino acids in the disallowed region, and we observed similar Ramachandran plot distributions in our previous study as well.[9]

**g) Preparation of DDM and peptides for testing on cancer cells**

Lyophilized DpPorA, LpPorA, DpPorA DE, LpPorA DE and 5-FAM-DpPorA DE were reconstituted in 0.1% DDM [1 µL of 100 µg $mL^{-1}$ DDM (Sigma-Aldrich Merck, D5172) in PBS (Gibco - 14040)], resulting in a peptide concentration of 1.7 mM. Subsequent concentrations were diluted with DMEM, 10% FBS, and 1% Penicillin /Streptomycin-containing media. **(Supplementary Table 2)**

**h) Cell membrane integrity analysis and statistics**

Immunofluorescence (IF) images were analyzed to assess the impact of different treatments on cell membrane integrity. Cells were categorized into Intact and Disrupted groups based on membrane integrity observed by CellMask cell membrane staining. We evaluated at least 100 cells for each group - Control, DDM-treated cells and peptide-treated cells- with independent analysis done by a student blinded to the groups' identity.

Cells in the "Intact" category displayed a well-defined membrane. The "Disrupted" category consisted of cells that exhibited membrane pore formation and internalization of the dye, indicating a loss of cell membrane integrity. This categorization provided a clear and systematic approach to quantify the effects of various treatments on cell membrane integrity.

For three independent experiments, data in bar graphs represent mean (± standard deviation) fold change relative to Control groups. Differences between the two datasets were assessed using Welch's test. Statistical significance is indicated as: *$p<0.05$, **$p<0.01$, ***$p<0.001$, ****$p<0.0001$.

## Supplementary figures:

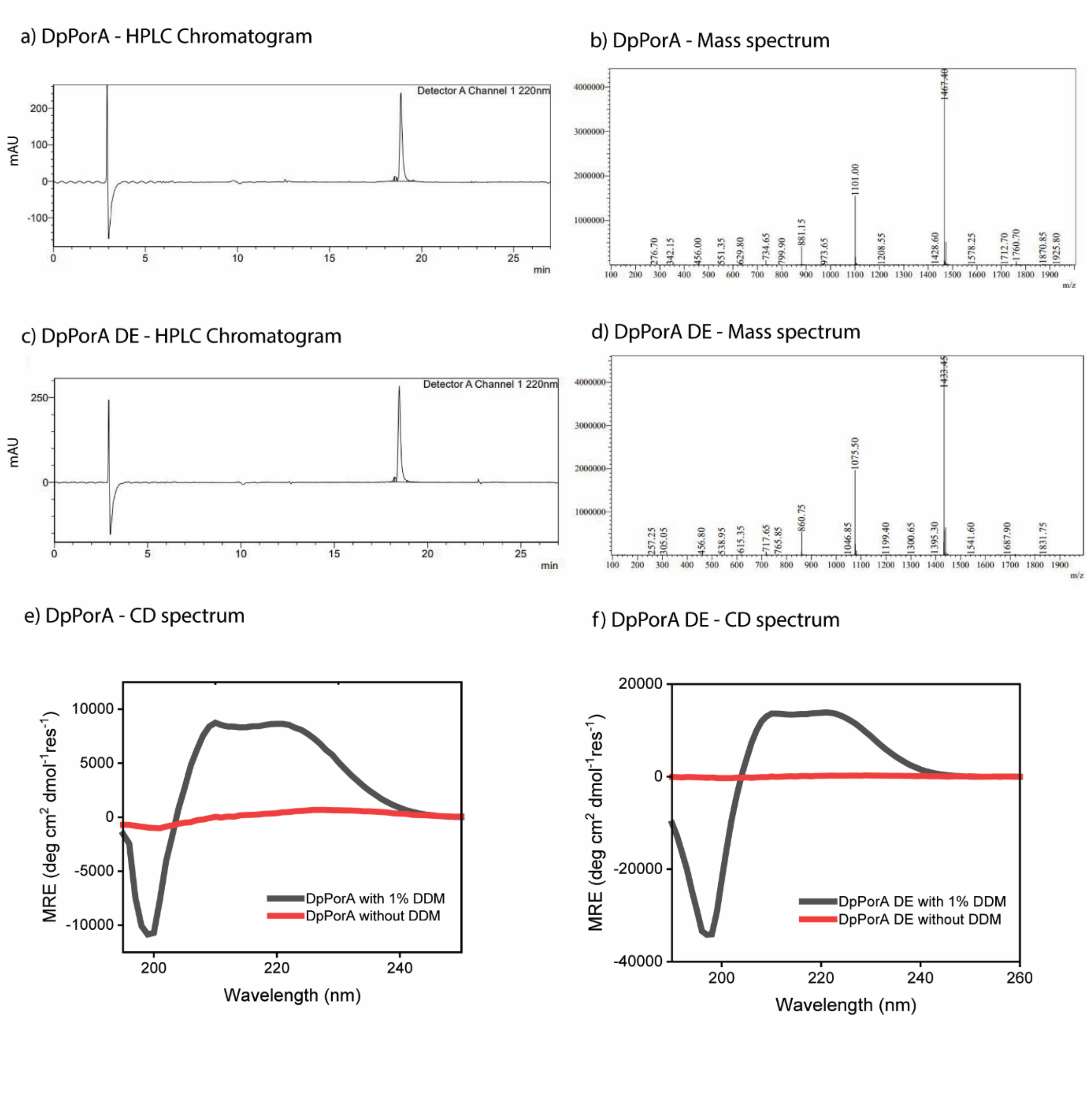


**Supplementary Fig. 1: HPLC traces, mass and CD spectra for DpPorA peptides.**

**a.** DpPorA HPLC traces (absorbance at 220 nm). **b.** DpPorA mass spectra observed mass - 4401.20 Da. **c.** DpPorA DE HPLC traces (absorbance at 220 nm). **d.** DpPorA DE mass spectra observed mass - 4299.15 Da. **e.** CD spectra of DpPorA with 1% DDM (black) and without DDM (red). **f.** CD spectra of DpPorA DE with 1% DDM (black) and without DDM (red).

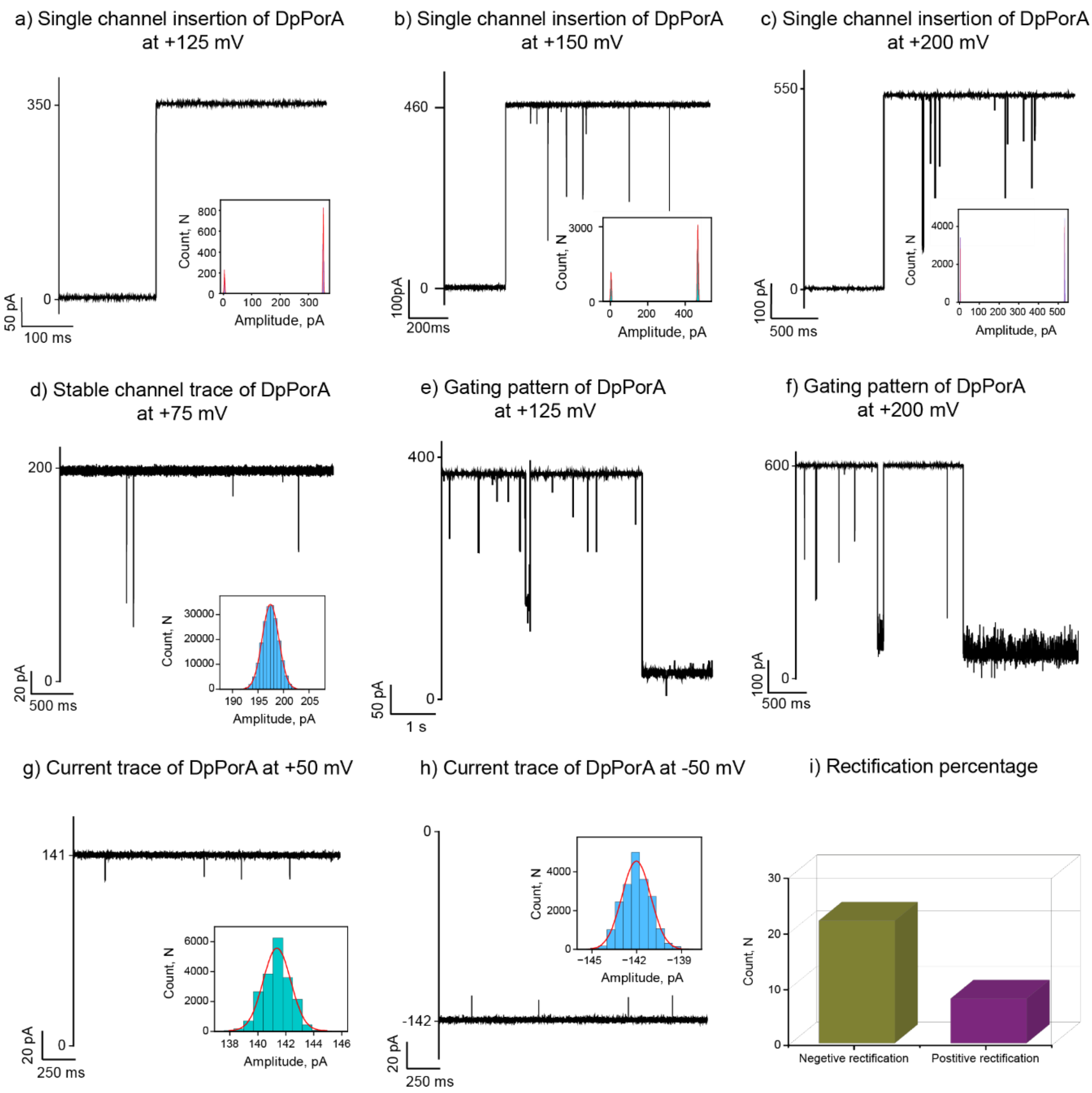


**Supplementary Fig. 2: Single-channel characterization of DpPorA.**

**a.** Single-channel insertion of DpPorA at +125 mV. **b.** Single-channel insertion of DpPorA at +150 mV. **c.** Single-channel insertion of DpPorA at +200 mV. Insets show the corresponding current-amplitude histogram. **d.** Stable channel trace of DpPorA at +75 mV. The inset shows the corresponding current-amplitude histogram. **e.** The current trace shows the gating pattern of DpPorA at +125 mV. **f.** The current trace shows the gating pattern of DpPorA at +200 mV. **g.** The current trace shows a slightly lower current at +50 mV than at **h.** -50 mV. **i.** The plot shows the most preferred orientation of DpPorA identified by the current rectification of n = 30 single-channels. The current signals were digitally filtered at 2 kHz. Electrolyte: 1 M KCl, 10 mM HEPES, pH 7.4.

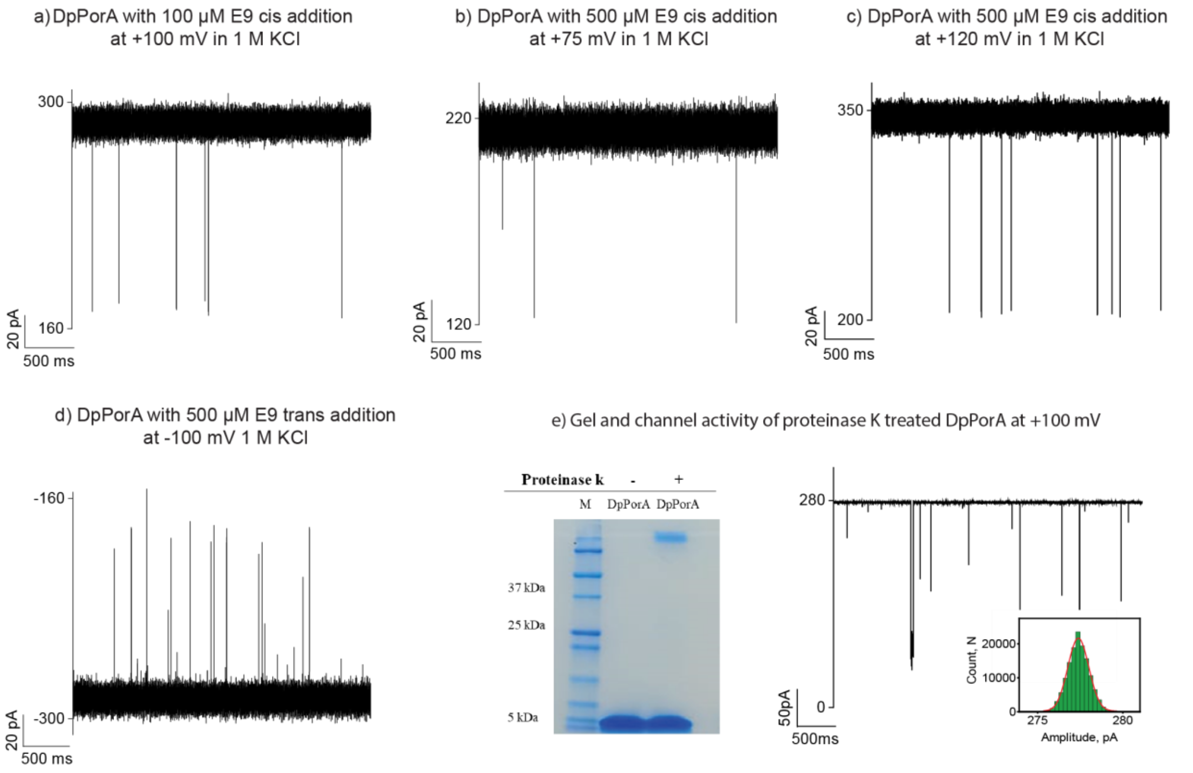


**Supplementary Fig. 3: Interaction of DpPorA with E9 peptides.**

**a.** Interaction of DpPorA with 100 µM E9 addition on the cis side at +100 mV. **b.** Interaction of DpPorA with 500 µM E9 addition on the cis side at +75 mV. **c.** Interaction of DpPorA with 500 µM E9 addition on the cis side at +120 mV. **d.** Interaction of DpPorA with 500 µM E9 addition on the trans side at -100 mV. **e.** Proteinase K treated, untreated DpPorA peptides run on SDS-PAGE showing monomer band. Data are representative of more than three repeats. Single stable pore insertion of proteinase K treated DpPorA peptides with stable conductance at +100 mV and corresponding current amplitude histogram. The current signals were filtered at 10 kHz and sampled at 50 kHz except for **(e)**. The current signal **(e)** was digitally filtered at 2 kHz. Electrolyte: 1 M KCl, 10 mM HEPES, pH 7.4.

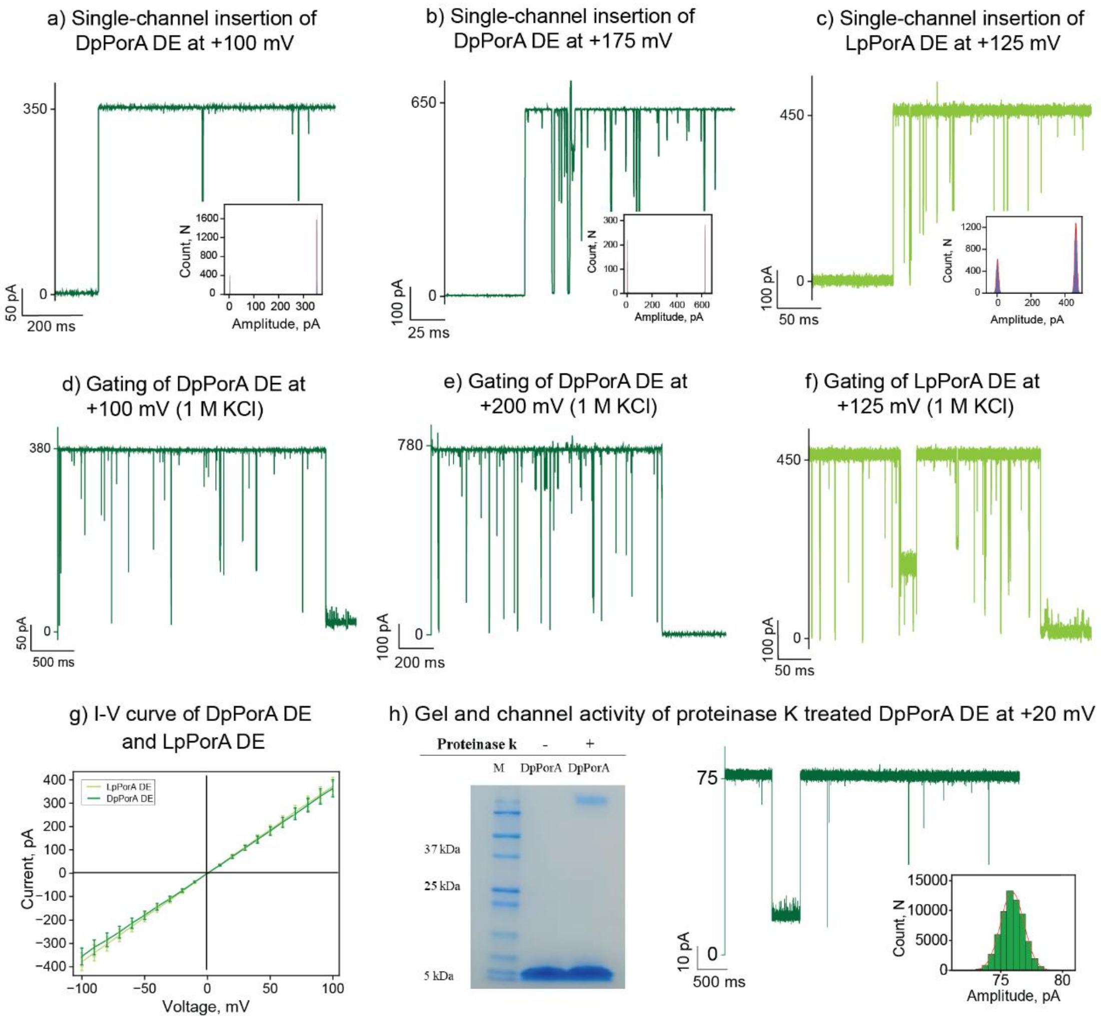


**Supplementary Fig. 4: Single-channel characterization of DpPorA DE.**

**a.** Single-channel insertion of DpPorA DE at +100 mV with corresponding current-amplitude histogram as inset. **b.** Single-channel insertion of DpPorA DE at +175 mV with corresponding current-amplitude histogram as inset. **c.** Single-channel insertion of LpPorA DE at +125 mV with corresponding current-amplitude histogram as inset. **d.** Gating closure of DpPorA DE at +100 mV. **e.** Gating closure of DpPorA DE at +200 mV. **f.** Gating closure of LpPorA DE at +125 mV. **g.** Overlapped I-V curve of DpPorA DE and LpPorA DE showing stable current from -100 mV to +100 mV. Error bars represent 10% standard error mean between 4 independent experiments. **h.** Proteinase K treated and untreated DpPorA DE peptides run on SDS-PAGE showing monomer band (data are representative of more than three repeats) and ion current trace showing proteinase K treated DpPorA DE at +20 mV. The inset shows the corresponding current-amplitude histogram. The current signals were digitally filtered at 2 kHz. Electrolyte: 1 M KCl, 10 mM HEPES, pH 7.4.

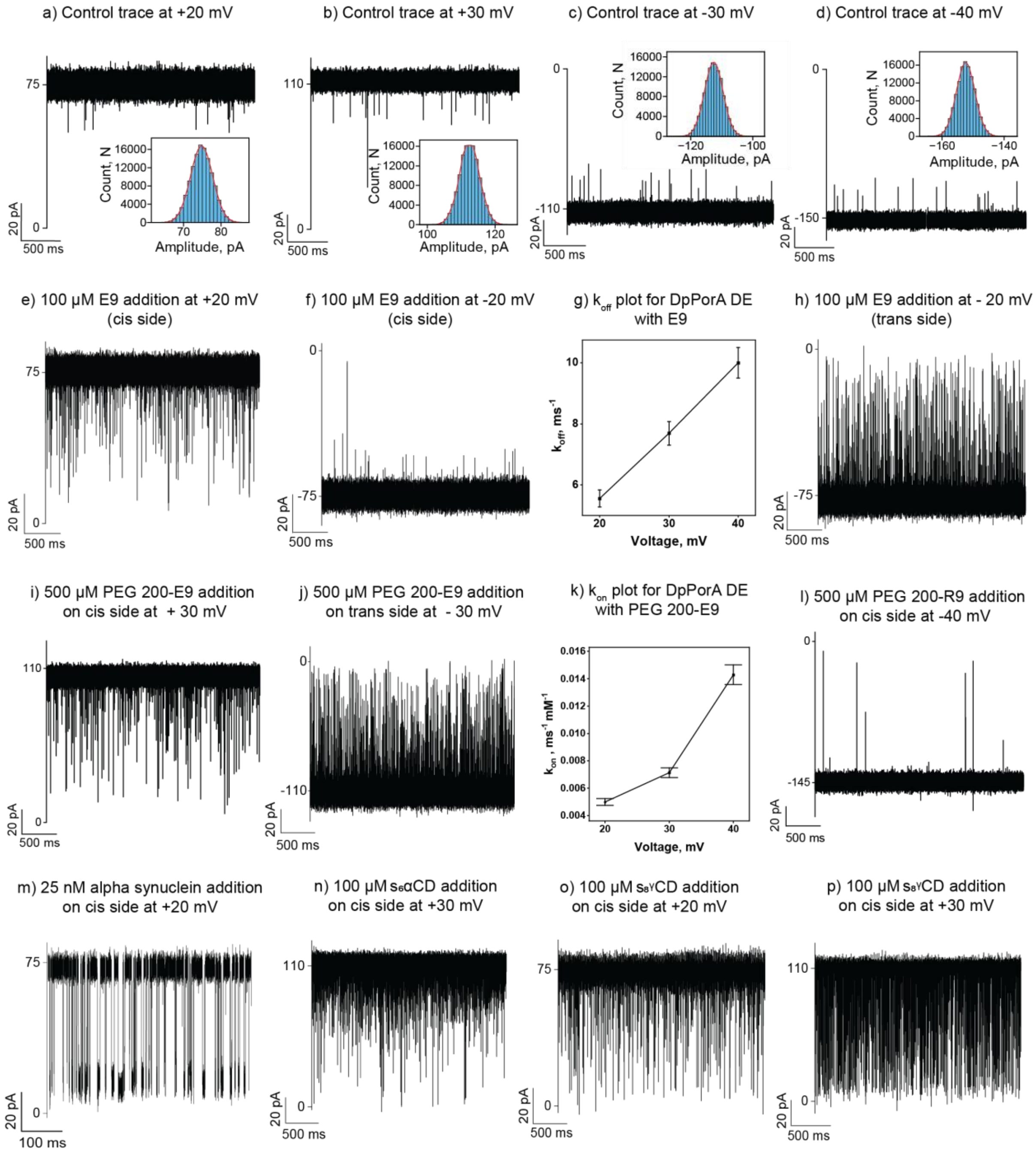


**Supplementary Fig. 5: Interaction of DpPorA DE without and with analytes.**

Control ion current traces of DpPorA DE at **a.** +20 mV, **b.** +30 mV, **c.** -30 mV and **d.** -40 mV, with corresponding current-amplitude histogram as inset. **e.** Ion current trace showing 100 µM E9 addition on the cis side of DpPorA DE at +20 mV. **f.** Due to electrophoretic repulsion, the electrical recording shows no interaction at -20 mV on 100 µM E9 addition on the cis side of DpPorA DE **g.** $k_{off}$ plots of E9 addition with DpPorA DE at three different voltages. Error bars represent 5% standard error mean between 3 independent experiments. **h.** Interaction of 100 µM E9 with DpPorA DE on the trans side at -20 mV. **i.** Ion current trace showing 500 µM PEG

200-E9 addition on the cis side of DpPorA DE at +30 mV. **j.** Ion current trace showing 500 μM PEG 200-E9 addition on the trans side of DpPorA DE at -30 mV. **k.** $k_{on}$ plots of PEG-200 E9 addition with DpPorA DE at three different voltages. Error bars represent 5% standard error mean between 3 independent experiments. **l.** Trace showing no interaction of DpPorA DE with 500 μM PEG 200-R9 on the cis side at -40 mV. **m.** Interaction of 25 nM alpha-synuclein (αS) with DpPorA DE on the cis side at +20 mV. **n.** Interaction of 100 μM anionic $s_6\alpha$CD with DpPorA DE on the cis side at +30 mV. Interaction of 100 μM anionic $s_8\gamma$CD with DpPorA DE on the cis side at **o.** +20 mV and **p.** +30 mV. The current signals were filtered at 10 kHz and sampled at 50 kHz. Electrolyte: 1 M KCl, 10 mM HEPES, pH 7.4.

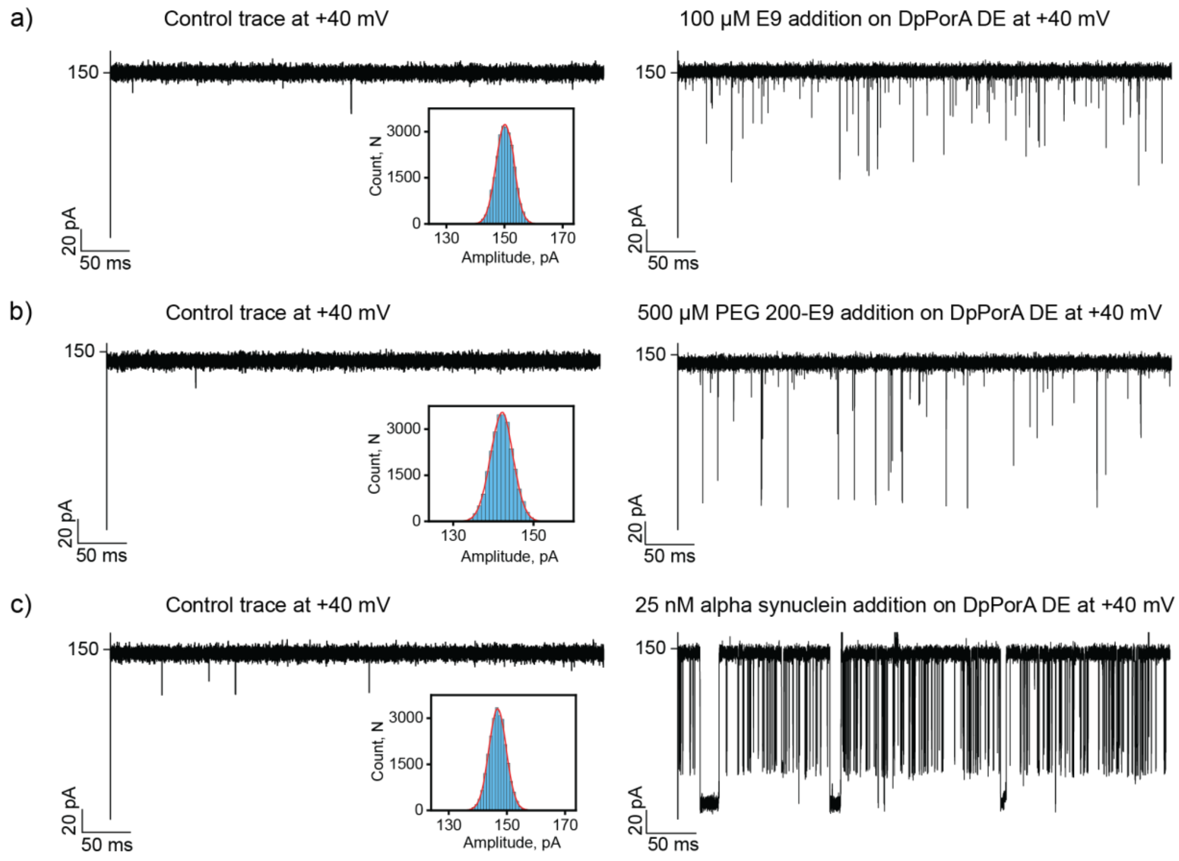


**Supplementary Fig. 6: Electrical recordings of DpPorA DE without and with analytes.**
**a.** Electrical recording of DpPorA DE without and with 100 µM E9 added to the cis side of the pore at +40 mV. **b.** Electrical recording of DpPorA DE without and with 500 µM PEG 200-E9 added to the cis side of the pore at +40 mV. **c.** Electrical recording of DpPorA DE without and with 25 nM alpha-synuclein (αS) added to the cis side of the pore at +40 mV. The current signals were filtered at 10 kHz and sampled at 50 kHz. Electrolyte: 1 M KCl, 10 mM HEPES, pH 7.4.

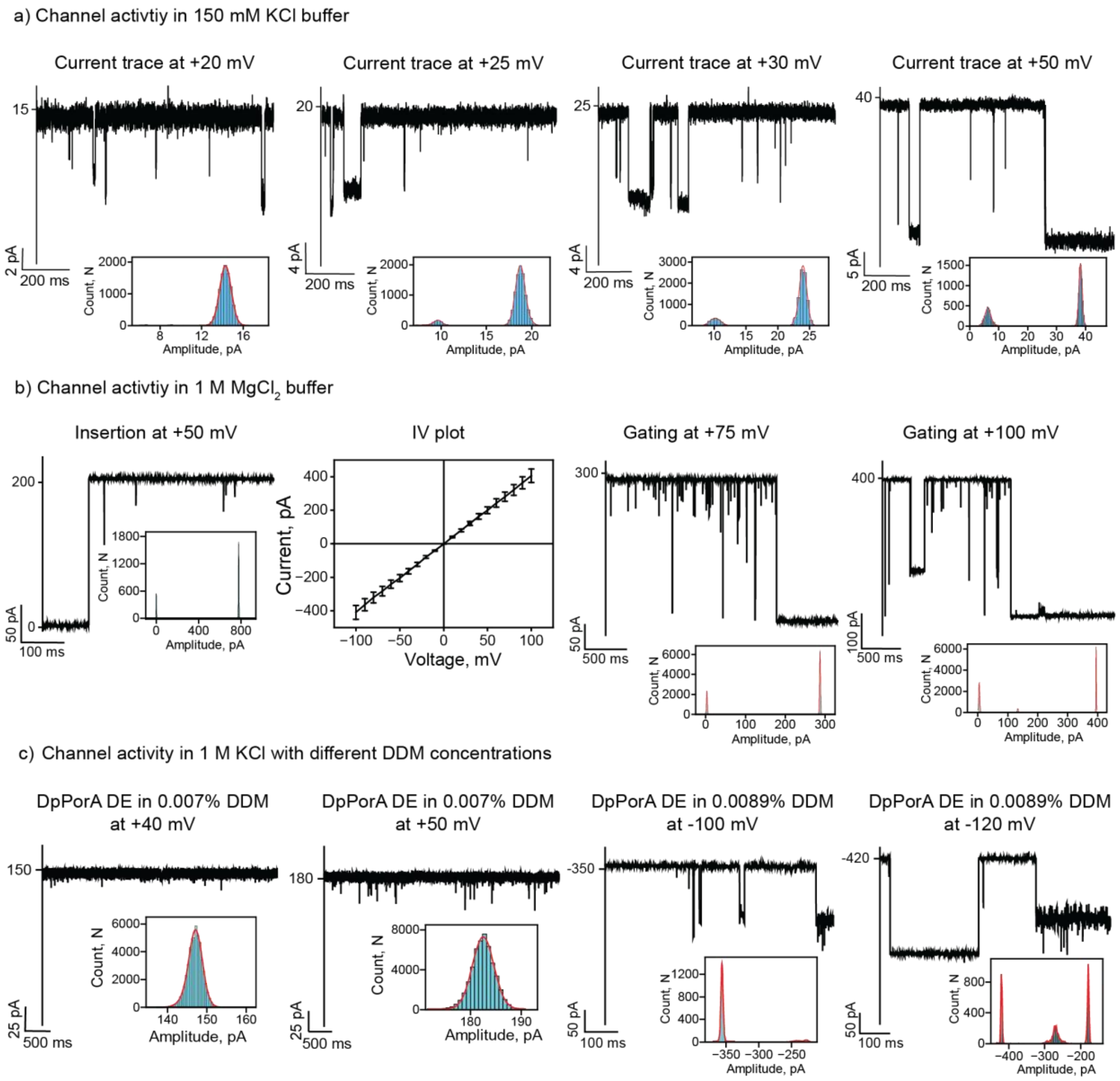


**Supplementary Fig. 7: Single-channel properties of DpPorA DE in different salt conditions and DDM concentrations.**

**a.** Single-channel electrical recording of DpPorA DE in 150 mM KCl buffer, 10 mM HEPES, pH 7.4. **b.** Single-channel electrical recording of DpPorA DE in 1 M $MgCl_2$ buffer, 10 mM HEPES, pH 7.4. Error bars represent 10% standard error mean between 4 independent experiments. **c.** Single-channel electrical recording of DpPorA DE with different DDM concentrations in 1 M KCl buffer, 10 mM HEPES, pH 7.4. The current signals were digitally filtered at 2 kHz.

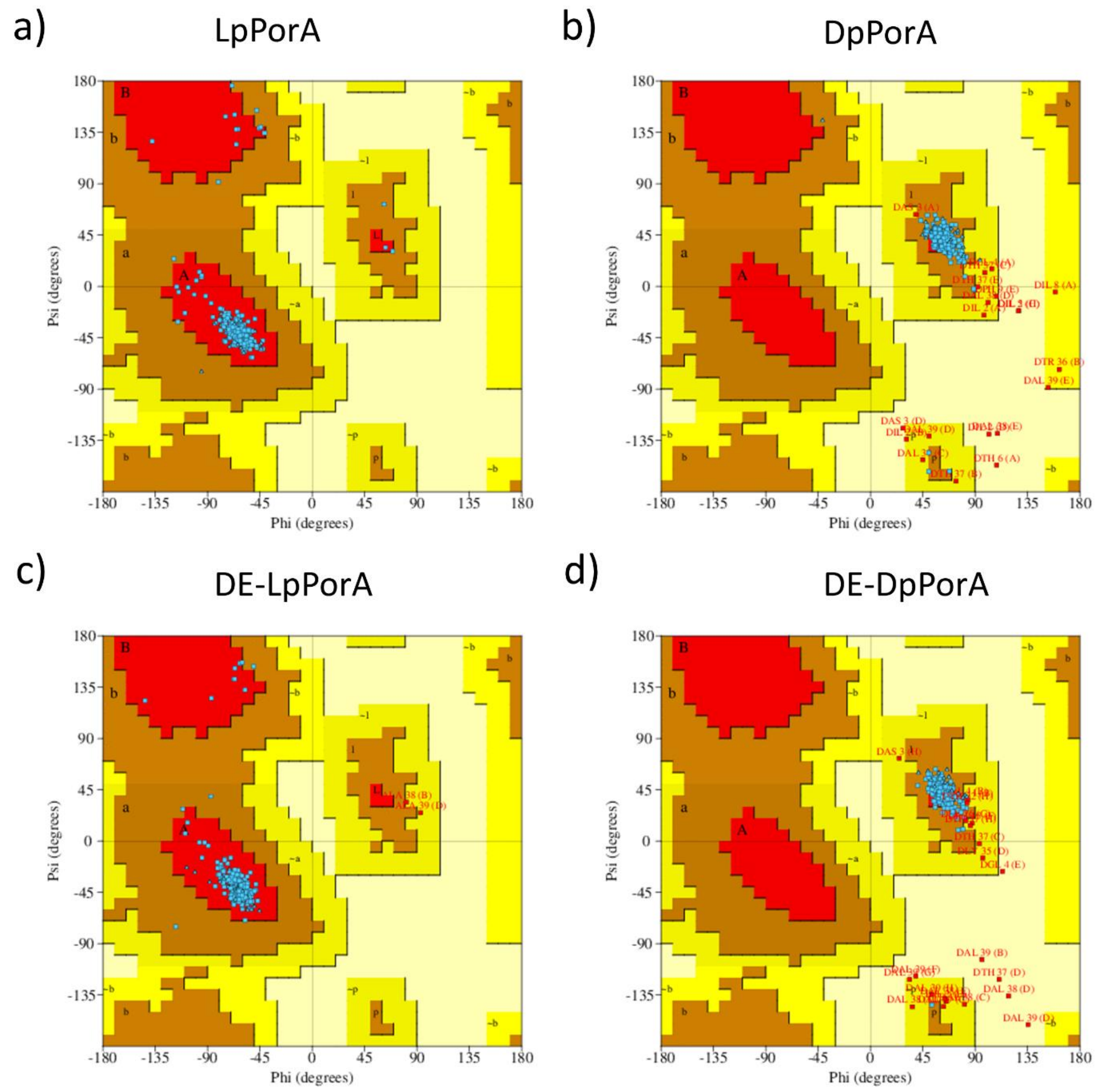


**Supplementary Fig. 8: Ramachandran plots of the pores.**

Ramachandran plots were generated using the PROCHECK web server for **a.** LpPorA, **b.** DpPorA, **c.** LpPorA DE, and **d.** DpPorA DE. The phi-psi torsion angles for all residues in the model structures are depicted in the figure by blue dots. The red areas correspond to the regions representing the expected most favorable combinations of phi-psi values. The region "A" is expected for right-handed α-helices, and region "L" for left-handed α-helices.

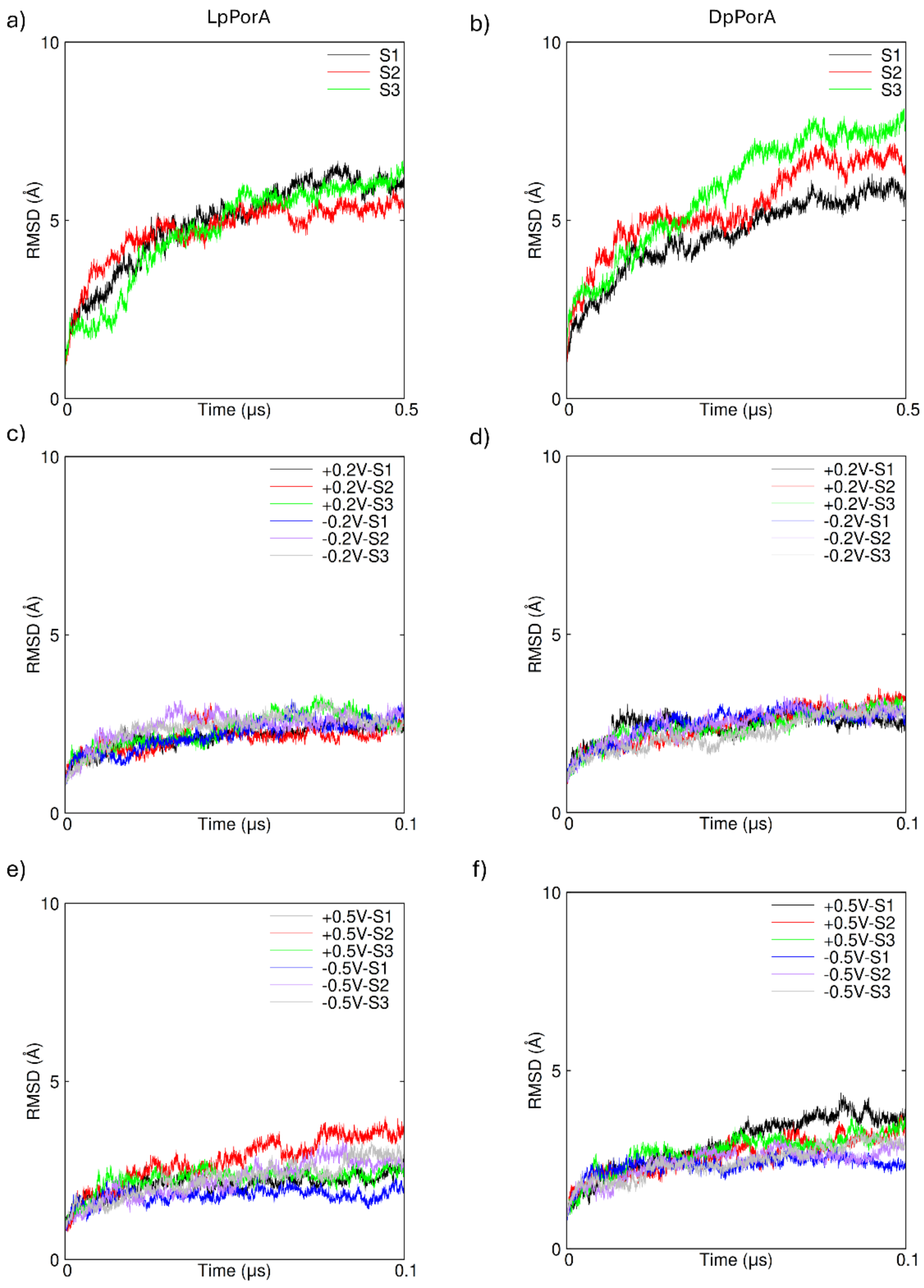


**Supplementary Fig. 9: Protein backbone-backbone RMSD values relative to the equilibrated pore structures of LpPorA and DpPorA.**

**a.** LpPorA and **b.** DpPorA RMSD distributions obtained from 500 ns-long unbiased MD simulation. Protein backbone-backbone RMSD for applied field MD simulations with respect to the structures obtained from 100 ns unbiased MD simulations. RMSD values for **c.** LpPorA and **d.** DpPorA based on 0.2 V applied-field simulations. **e.** and **f.** The same as c) and d) but at 0.5 V.

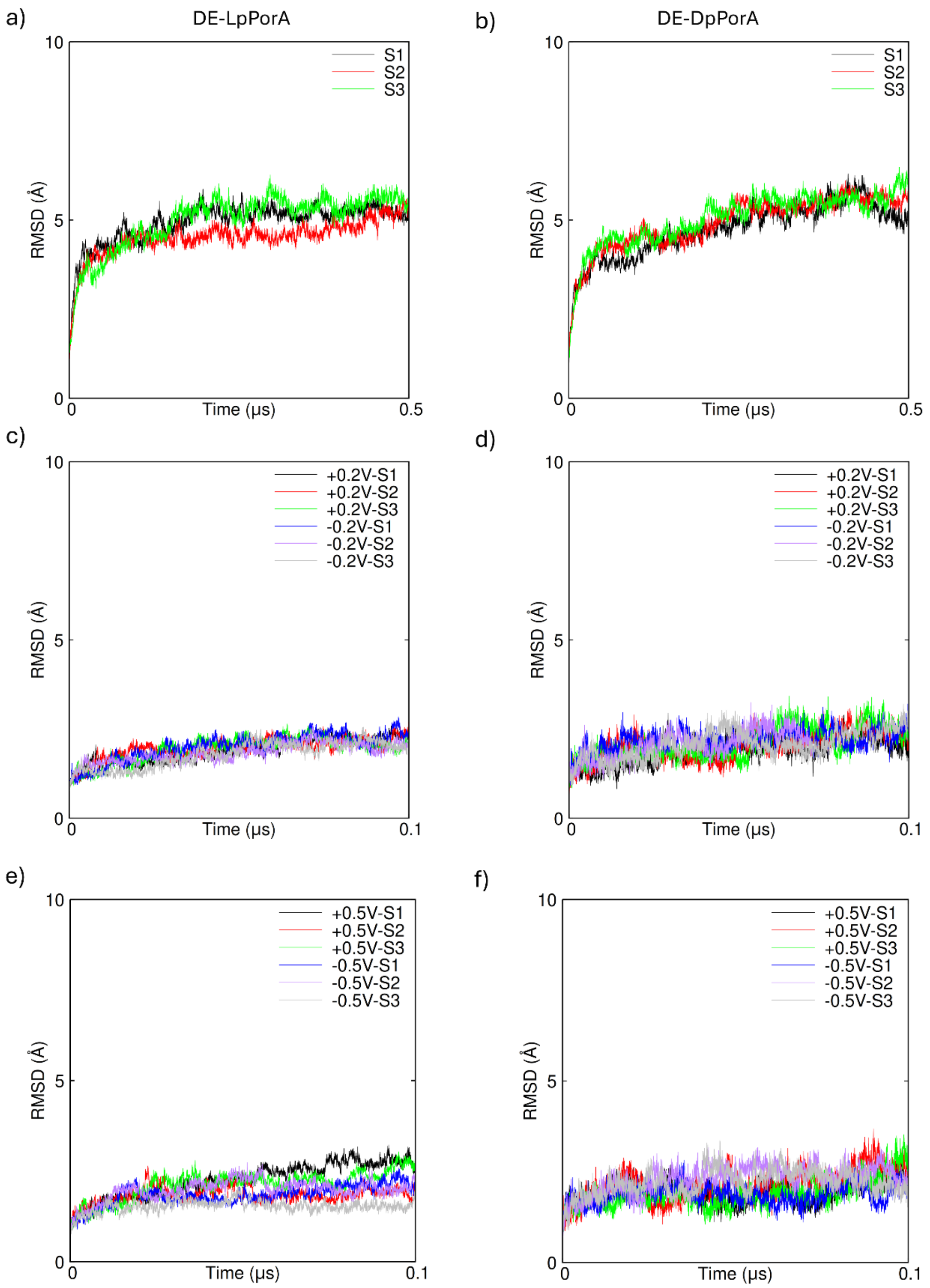


**Supplementary Fig. 10: Protein backbone-backbone RMSD values relative to the equilibrated pore structures of LpPorA DE and DpPorA DE.**

**a.** LpPorA DE and **b.** DpPorA DE RMSD distributions obtained from 500 ns-long unbiased MD simulation. Protein backbone-backbone RMSD for applied field MD simulations with respect to the structures obtained from 100 ns unbiased MD simulations. RMSD values for **c.** LpPorA DE and **d.** DpPorA DE based on 0.2 V applied-field simulations. **e.** and **f.** The same as c) and d) but at 0.5 V.

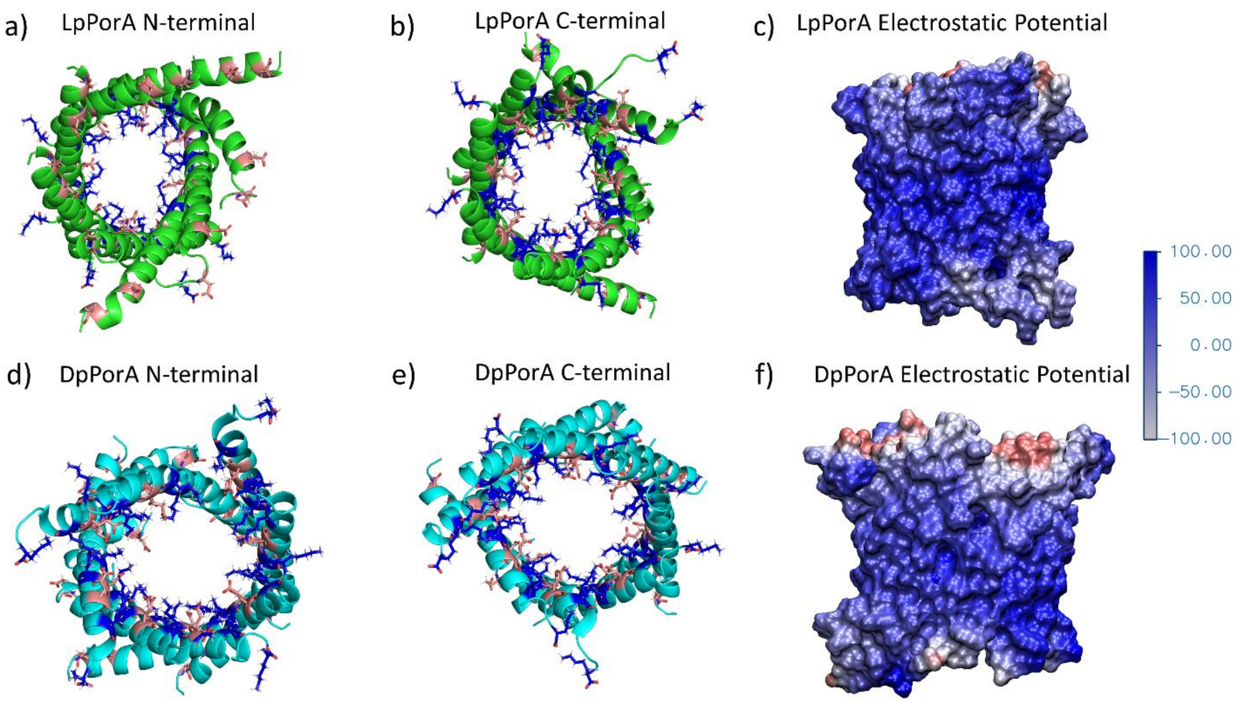


**Supplementary Fig. 11: Structures and electrostatic potentials of LpPorA and DpPorA.** **a. b.** N-terminal and C-terminal views of LpPorA structures are depicted in the green cartoon at the end of the 500 ns-long unbiased simulation. Basic amino acid residues are depicted in blue, while acidic residues are depicted in salmon-red. **c.** Side view of the electrostatic protein of LpPorA. **d-f.** Same as a-c but for DpPorA.

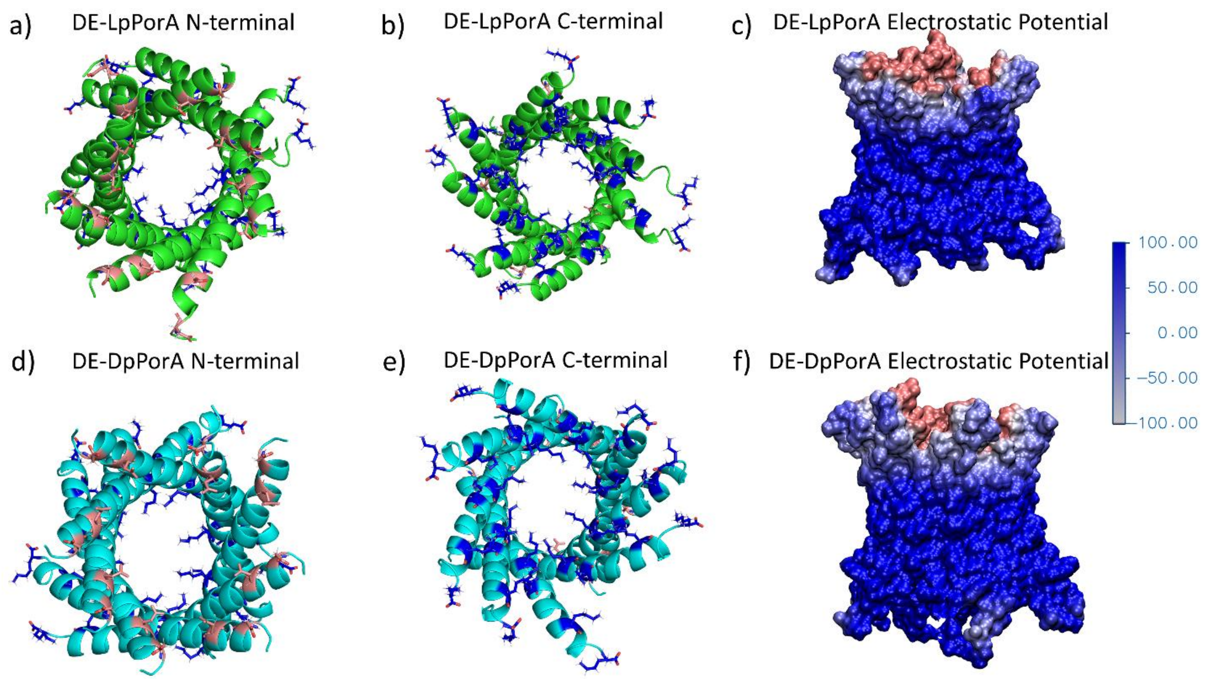


**Supplementary Fig. 12: Structures and electrostatic potentials of LpPorA DE and DpPorA DE.**

**a. b.** N-terminal and C-terminal views of LpPorA DE structures depicted in green cartoon representation based on the 500 ns-long unbiased simulation. Basic amino acid residues are depicted in blue, while acidic residues are depicted in salmon-red. **c.** Side view of the electrostatic protein of LpPorA DE. **d-f.** Same as **a-c** but for DpPorA DE.

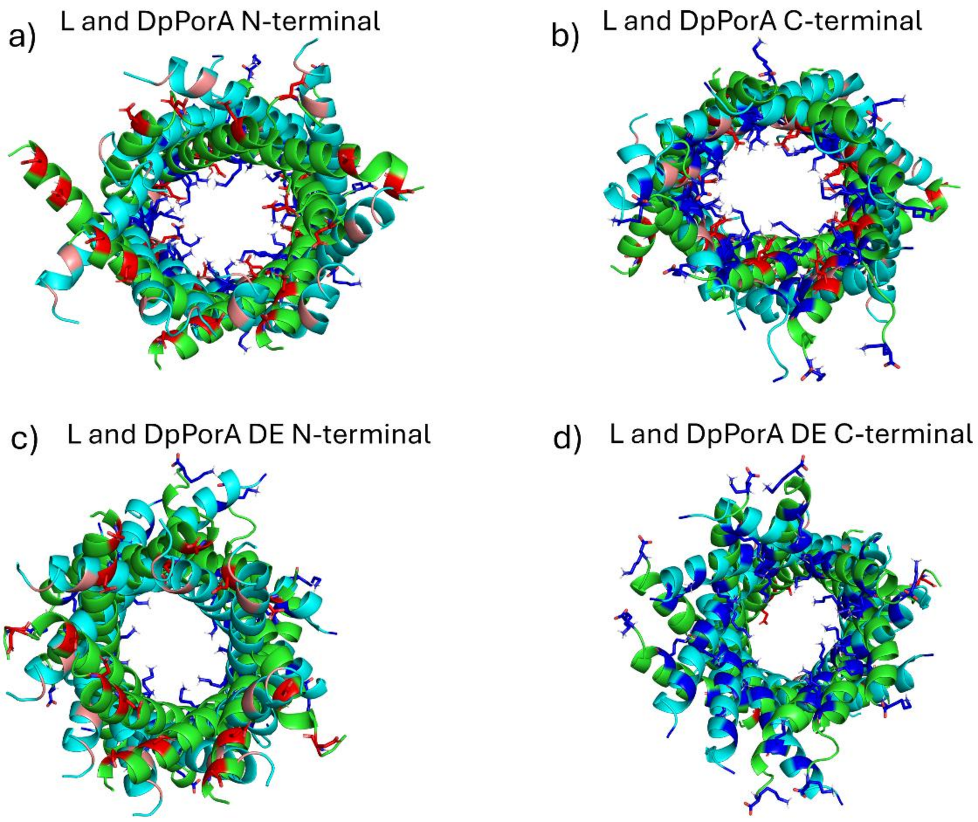


**Supplementary Fig. 13: Overlapped structure view of L and D pores.**

**a. b.** N-terminal and C-terminal overlapped views of LpPorA (green) and DpPorA (cyan) structures in the cartoon at the end of the 500 ns-long unbiased simulation. Basic amino acid residues are represented in blue, while acidic residues are shown in red. **c. d.** Same as a-b but for L and DpPorA DE.

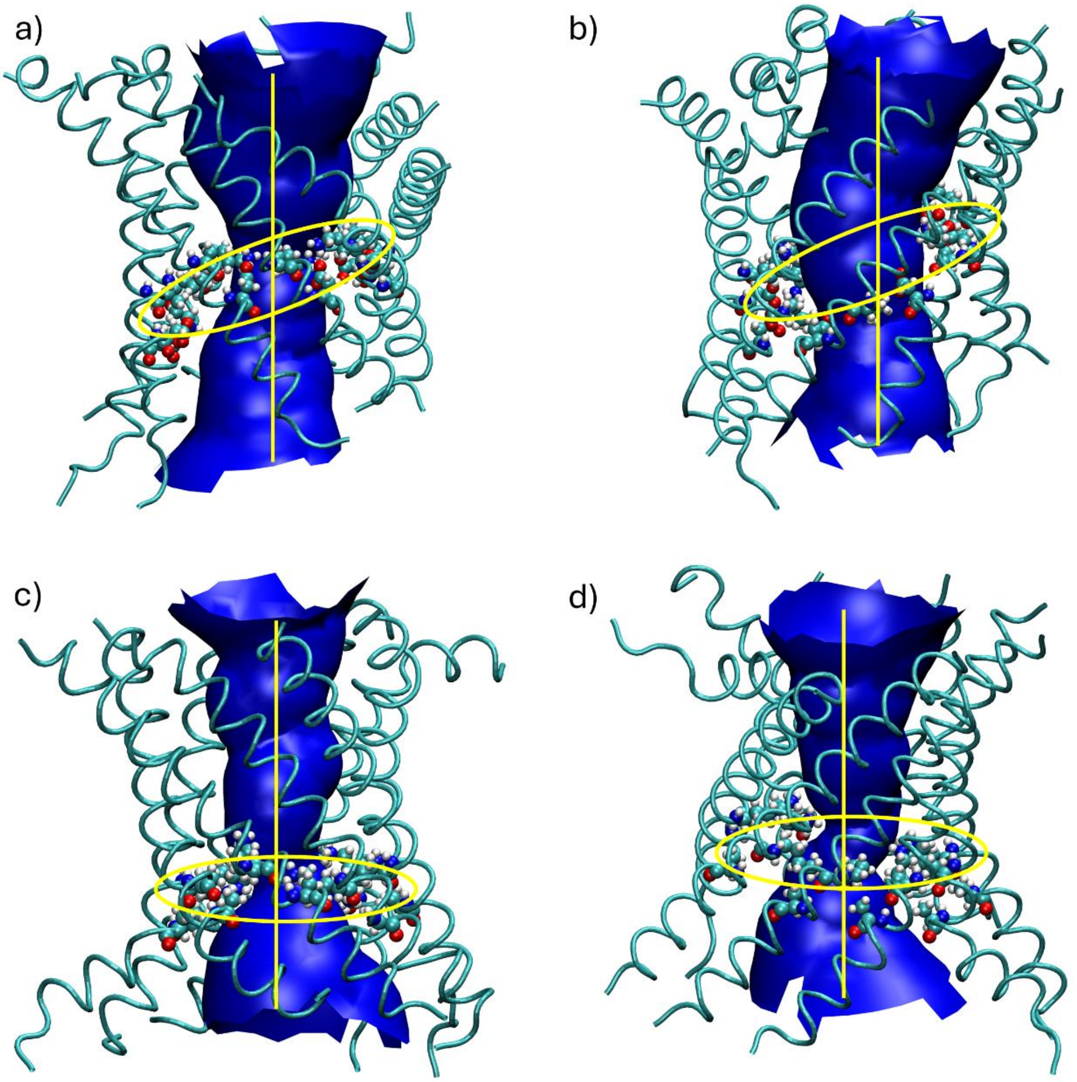


**Supplementary Fig. 14**: **The HOLE surface-radius profile along with the designed pore.** **a.** The surface HOLE profile is generated from the 500 ns final structure of the LpPorA pore. The angled orientation of the salt bridge interacting residues LYS24 and ASP28 is depicted with a "CPK model," where the pore is shown with a tube. **b.** The surface hole profile of DpPorA. **c.** The surface hole profile of LpPorA DE. The absence of the salt bridge interactions allowed the LYS24 residues to be oriented perpendicular to that of the Z-axis. **d.** A similar HOLE radius profile was observed for the DpPorA DE pore.

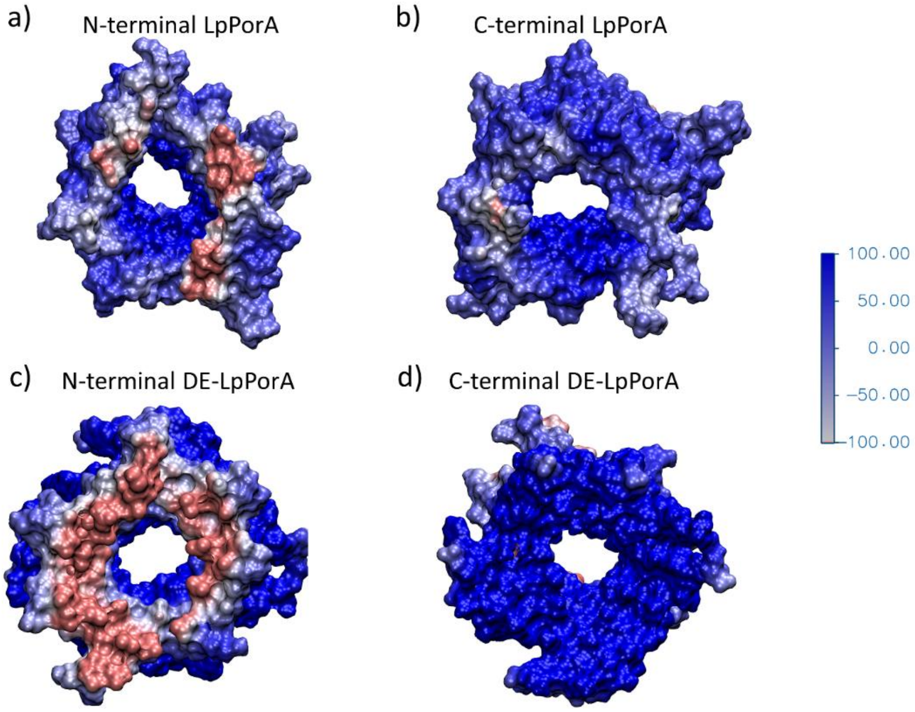


**Supplementary Fig. 15: Electrostatic potential maps of LpPorA and LpPorA DE.**

**a. b.** N-terminal and C-terminal views of the electrostatic potential map showing the amino residues of LpPorA lining the interior channel wall. The computed electrostatic potential ranges from −198 to +313 $k_BT/e$ for LpPorA, where 1 $k_BT/e$ equals 26 mV at 300 K. To enhance clarity, the color range for the electrostatic potential has been restricted from −100 to +100 $k_BT/e$. **c. d.** Same as a,b but for LpPorA DE. The computed electrostatic potential ranges from −272 to +815 $k_BT/e$ for LpPorA DE.

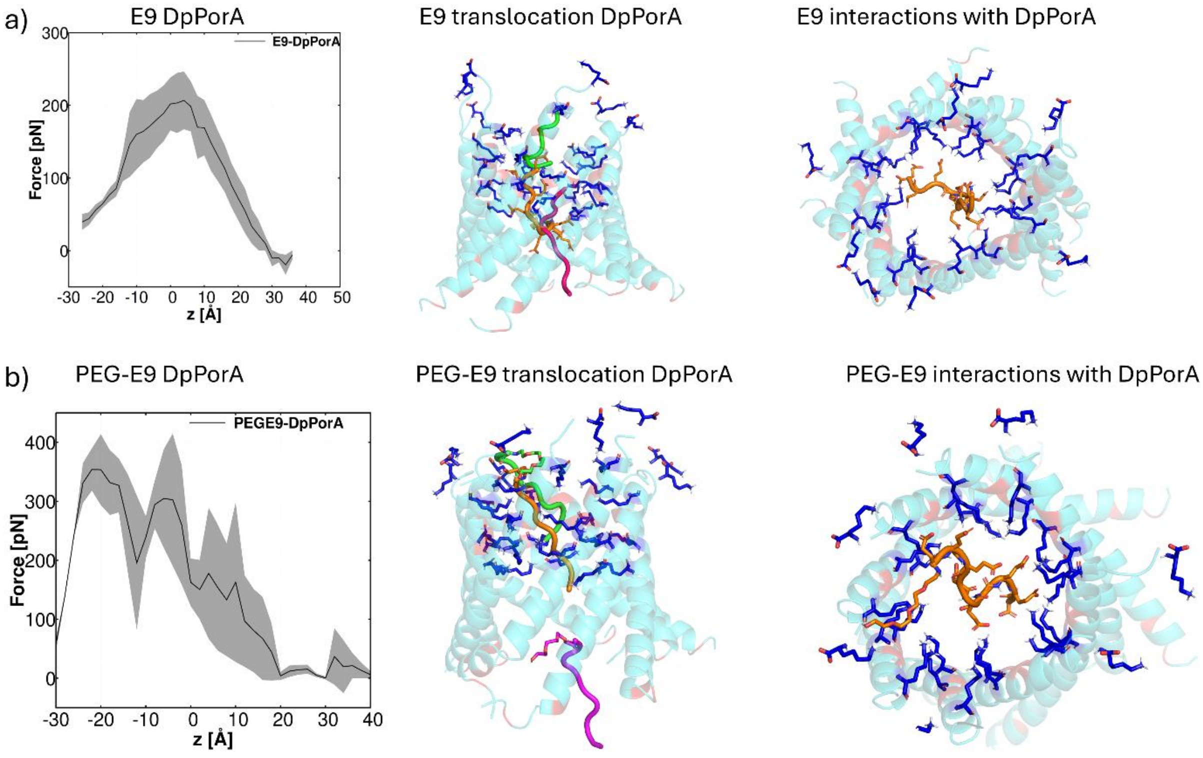


**Supplementary Fig. 16: Translocation of E9 and PEG 200-E9 through DpPorA.**

**a.** Average force profile from steered MD simulations depicting the permeation of anionic nonaglutamic acid as a function of reaction coordinate *z*. The average force profile is depicted as a bold line, and the standard deviation as a transparent shade. This coordinate corresponds to the center of the mass distance between nonaglutamic acid and the $C_\alpha$ atoms of DpPorA. The shaded error bars represent the standard deviations from the three simulations. In the middle panel, three different positions of E9 in the DpPorA are shown from the translocation pathway: the initial position (green), one in the middle of the pore corresponding to the peak position of the force profile (brown), and the final conformation (magenta) showing the E9 peptide leaving the interaction zone of DpPorA. The interactions between the negatively charged acidic groups of the E9 peptide and the NH3+ groups of the lysine residues along the translocation pathway through DpPorA are shown in the right part of the figure. **b.** Same as **a**, but for PEG 200-nonaglutamic acid as substrate. The PEG 200 moieties are shown as stick structures attached to the nonaglutamic acid peptide.

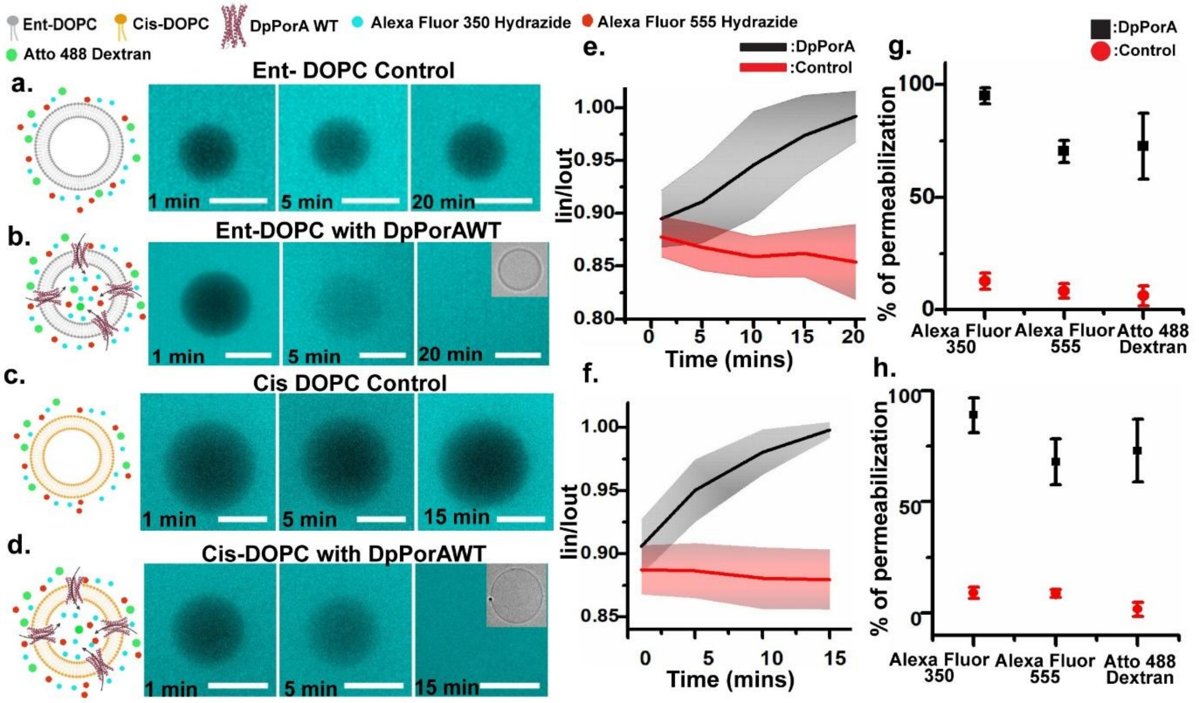


**Supplementary Fig. 17: Transport across DpPorA in Enantiomeric and Cis DOPC giant unilamellar vesicle systems.**

The images depicting the transport of Alexa Fluor 350 in **a.** Ent-DOPC vesicles with 0.1% DDM. **b.** Ent-DOPC incorporated with DpPorA **c.** Cis-DOPC vesicles with 0.1% DDM **d.** Cis-DOPC vesicles incorporated with DpPorA. Graph showing the time-dependent analysis of normalized intensities of individual **e.** Ent-DOPC vesicles with (n = 10) and without DpPorA (n = 5) **f.** Cis-DOPC vesicles with and without DpPorA (n = 8). Statistical analysis of the vesicle percentage for permeabilization of Alexa Fluor 350, Alexa Fluor 555, and ATTO 488 Dextran in **g.** Ent-DOPC vesicles incorporated with DpPorA (n = 123, 62, and 61 vesicles for Alexa Fluor 350, Alexa Fluor 555, and ATTO 488 Dextran, respectively from N = 4 batches) and control Ent-DOPC vesicles (n = 132, 60, and 72 vesicles for Alexa Fluor 350, Alexa Fluor 555, and ATTO 488 Dextran, respectively from N = 4 batches) and **h.** Cis-DOPC vesicles incorporated with DpPorA (n = 136, 64, and 72 vesicles for Alexa Fluor 350, Alexa Fluor 555, and ATTO 488 Dextran, respectively from N = 4 batches) and control Cis-DOPC vesicles (n = 123, 63, and 60 vesicles for Alexa Fluor 350, Alexa Fluor 555, and ATTO 488 Dextran, respectively from N = 4 batches). Permeabilization percentages for Ent-DOPC vesicles were 95.13±3.54 for Alexa Fluor 350, 70.38±4.93 for Alexa Fluor 555, and 72.70±14.61 for ATTO 488 dextran. Ent-DOPC control vesicles showed permeabilization of 12.69±3.63 for Alexa Fluor 350, 8.39±3.1 for Alexa 555 hydrazide, and 6.22±4.47 for ATTO 488 dextran.

Permeabilization percentages for Cis DOPC vesicles were 88.9±7.8 (Alexa Fluor 350), 67.9± 10.4 (Alexa Fluor 555), and 73.0 ± 14.1 (ATTO 488 Dextran). Cis DOPC control vesicles showed permeabilization of 9.08±2.56 for Alexa Fluor 350, 8.9±1.63 for Alexa 555 hydrazide, and 1.78±3.09 for Atto 488 dextran. The percentage of permeabilization was denoted as mean ± S.D. The number of batches and the total number of vesicles are denoted as 'N' and 'n', respectively. Buffer conditions: 100 mM KCl in 10 mM HEPES (pH: 7.4). Scale bar: 10 µm. Schematic figures of GUVs are created with BioRender.com.

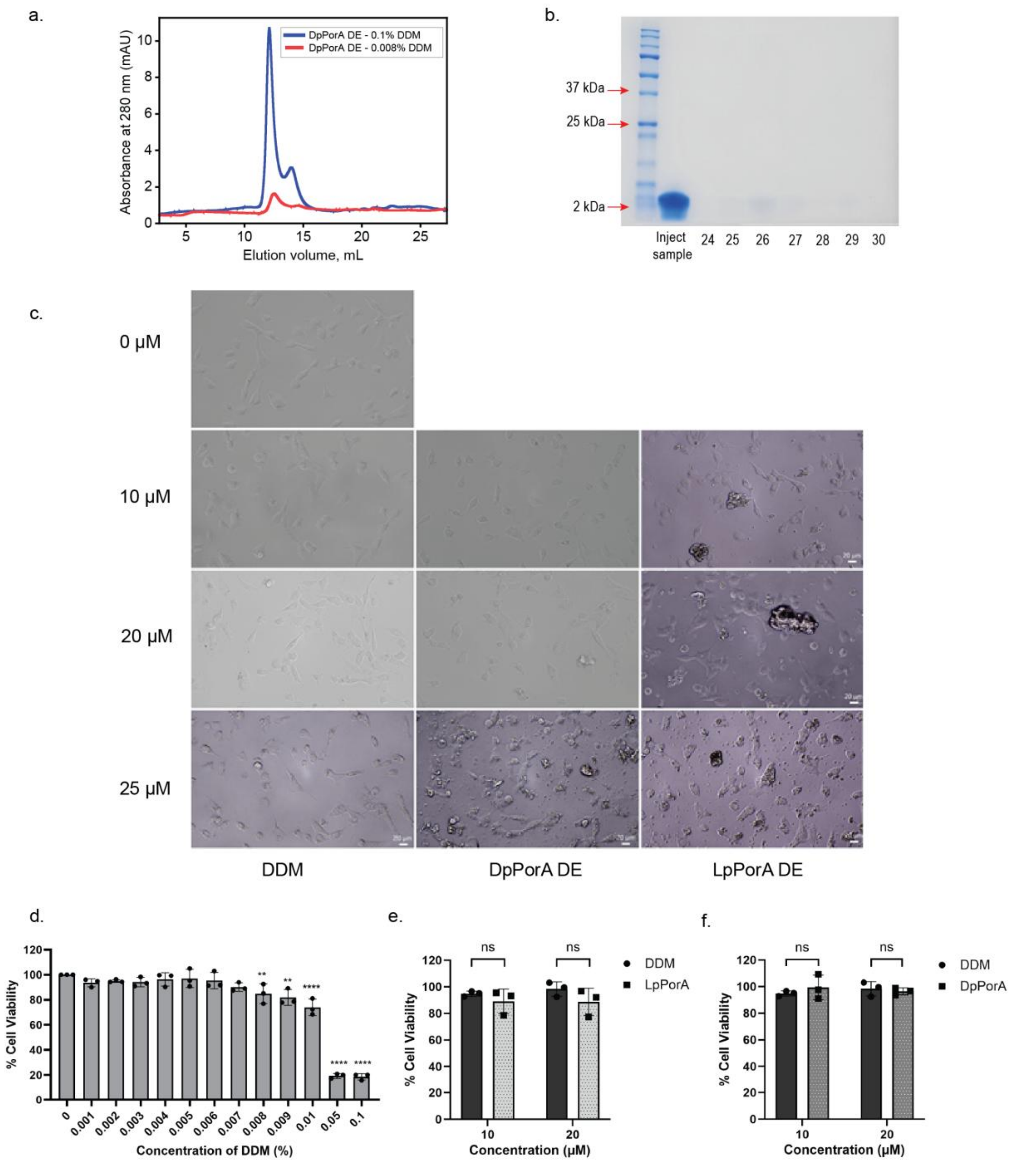


**Supplementary Fig. 18: Effect of DDM and pPorA peptides on MDA-MB-231 cells.**

**a.** Analytical SEC of DpPorA DE in 0.1% DDM and 0.008% DDM. **b.** SDS-PADE analysis of the SEC fractions collected between 11 mL and 16 mL (Fraction number 24 - 30) **c.** Microscopy images showing that peptide aggregation was not observed till 25 µM concentration used in the experiments (20X magnification) Scale bar: 20 µm. **d.** MDA-MB-231 cells were treated with DDM concentrations ranging from 0.001% to 0.1% for 24 hours, and cell viability was measured using the MTT assay. Viability decreased with increasing DDM concentrations. The

viability was comparable to control till 0.007% DDM, while concentrations above 0.007% showed significant cytotoxic effects. Concentrations below 0.007% are non-detrimental to cell viability. Data represents mean ± SEM from n = 3. Each dot represents a biological replicate, where each replicate was an independently seeded and treated culture of MDA-MB-231 cells on different days with different passage number. Statistical analysis was performed using one-way ANOVA with Dunnett's multiple comparisons test comparing each treatment to the untreated control (0 µM); Adjusted p values correspond to **p = 0.0080 (0.008% DDM), **p = 0.0012 (0.009% DDM), ****p = <0.0001 (0.01%-0.1% DDM). **e.** The % viability of MDA-MB-231 cells was determined using the MTT assay 24 hours after the addition of 10 µM and 20 µM LpPorA peptide and corresponding DDM. Data represents mean ± SEM from n = 3. Each dot represents a biological replicate, where each replicate was an independently seeded and treated culture of MDA-MB-231 cells on different days with different passage number. Statistical analysis was performed using ordinary two-way ANOVA with multiple comparison tests comparing LpPorA treatment to the DDM treatment; Adjusted p values correspond ns = non-significant, p = 0.7182 (10 µM) and p = 0.4411 (20 µM). **f.** The % viability of MDA-MB-231 cells was determined using the MTT assay 24 hours after the addition of 10 µM and 20 µM DpPorA peptide and corresponding DDM. Data represents mean ± SEM from n = 3. Each dot represents a biological replicate, where each replicate was an independently seeded and treated culture of MDA-MB-231 cells on different days with different passage number. Statistical analysis was performed using ordinary two-way ANOVA with multiple comparison tests comparing DpPorA treatment to the DDM treatment; Adjusted p values correspond ns = non-significant, p = 0.5919 (10 µM) and p = 0.8953 (20 µM).

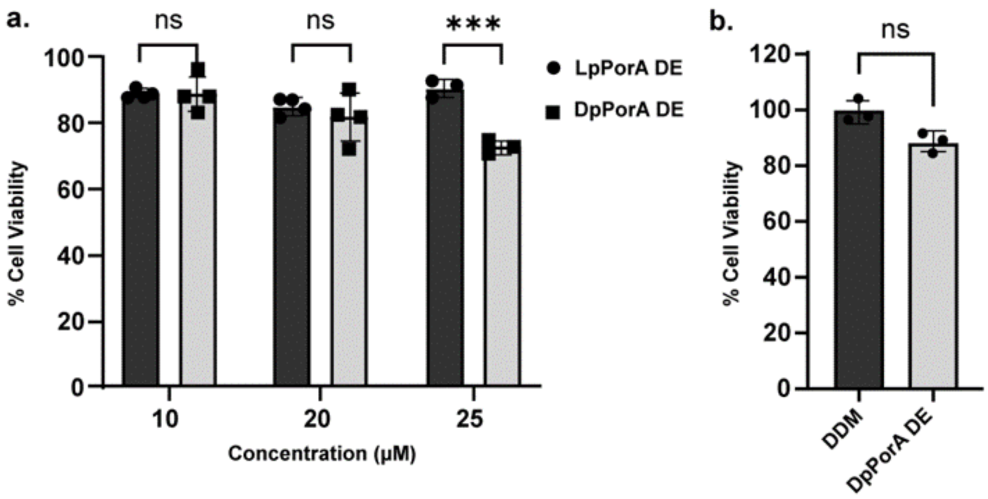


**Supplementary Fig. 19: Effect of DpPorA DE and LpPorA DE peptides on MDA-MB-231 and MCF10A cells.**

**a**. The % viability of MDA-MB-231 cells was determined using the MTT assay 24 hours after the addition of LpPorA DE and DpPorA DE peptides at 10 µM, 20 µM and 25 µM concentrations. Data represents mean ± SEM from n = 4 (10 µM and 20 µM) and n = 3 (25 µM). Each dot represents a biological replicate, where each replicate was an independently seeded and treated culture of MDA-MB-231 cells on different days with different passage number. Graph demonstrating a significant decrease in viability of cells treated with the DpPorA DE compared to LpPorA DE peptide in MDA-MB-231 cells. Statistical analysis was performed using two-way ANOVA with multiple comparison tests comparing effect of LpPorA DE to DpPorA DE on MDA-MB-231 cells and adjusted p values corresponds to ns = non significant, $p > 0.9999$ (10 µM), $p = 0.6386$ (20 µM) and ***$p = 0.0003$ (25 µM) . **b.** The % viability of MCF10A cells was determined using the MTT assay 24 hours after the addition of DpPorA DE peptides at 20 µM. Data represents mean ± SEM from n = 3. Each dot represents a biological replicate, where each replicate was an independently seeded and treated culture of MCF10A cells on different days with different passage number. Statistical analysis was performed using ordinary one-way ANOVA comparing the treatment to the DDM treatment showing no significant effect ($p = 0.1542$) of 20 µM DpPorA DE on MCF10A cells

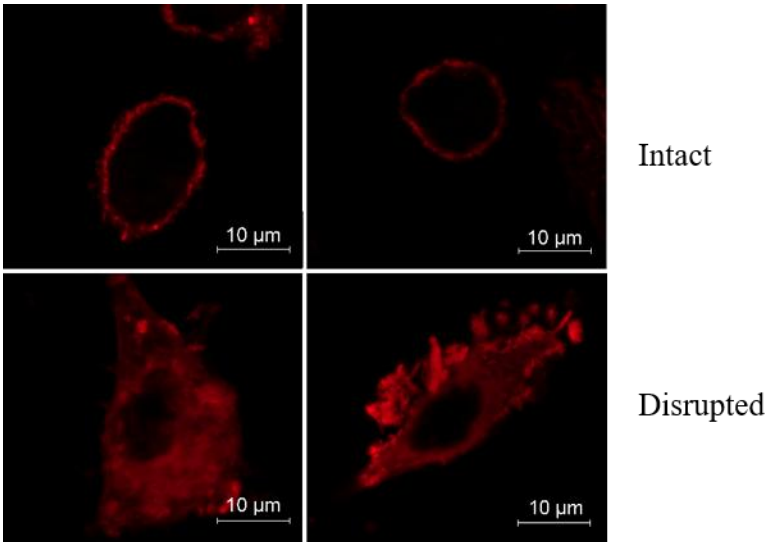


**Supplementary Fig. 20: Fluorescence microscopy images showing two cell membrane integrity phenotypes observed in the MDA-MB-231 cells.**

Representative images of Intact and Disrupted cells in 25 μM DpPorA DE peptide-treated MDA-MB-231 cells. (100 cells per group, scale bar 10 μm)

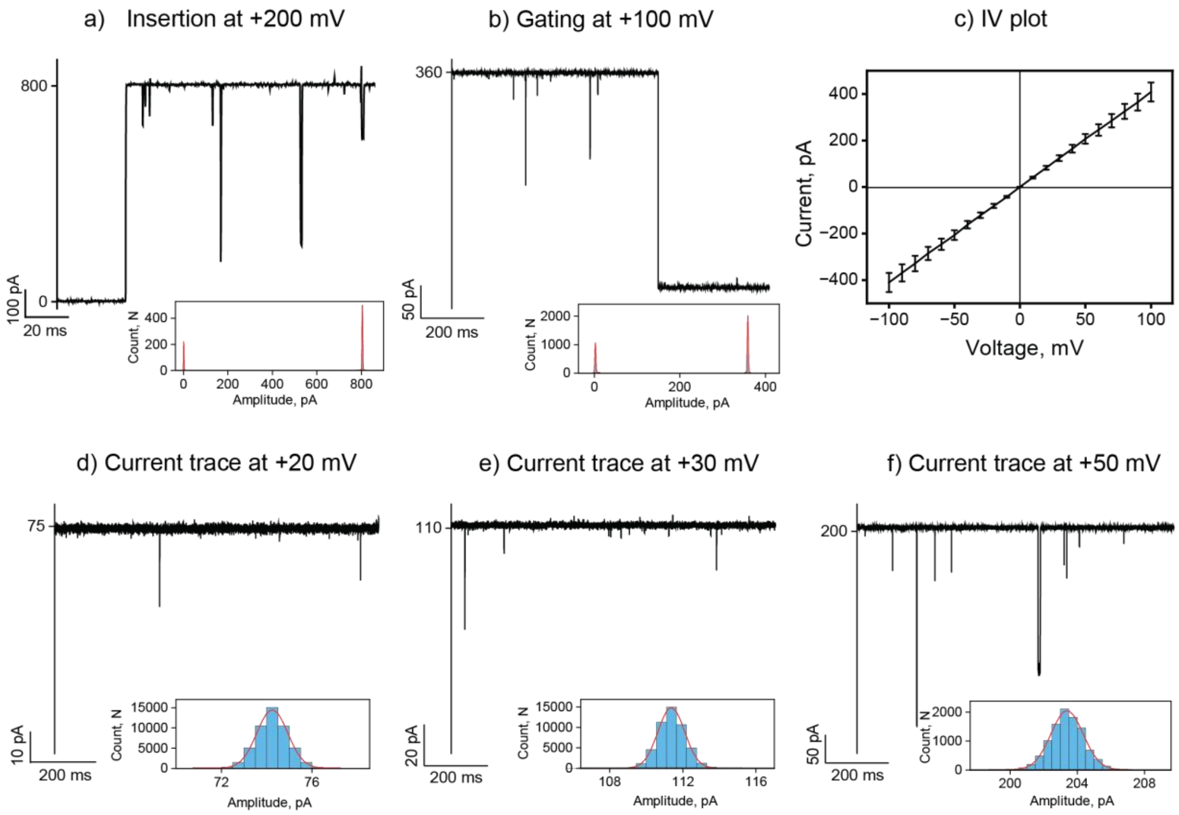


**Supplementary Fig. 21: Single-channel properties of 5-FAM-DpPorA DE.**

**a.** Single-channel insertion of 5-FAM-DpPorA DE at +200 mV, with corresponding current-amplitude histogram as inset. **b.** Electrical recording showing characteristic gating of 5-FAM-DpPorA DE at +100 mV, with corresponding current-amplitude histogram as inset. **c.** I-V curve of 5-FAM-DpPorA DE showing stable current from -100 mV to +100 mV. Error bars represent 10% standard error mean between 4 independent experiments. Electrical recording showing stable current trace of 5-FAM-DpPorA DE at **d.** +20 mV, **e.** +30 mV, and **f.** +50 mV, with corresponding current-amplitude histogram as inset. The current signals were digitally filtered at 2 kHz. Electrolyte: 1 M KCl, 10 mM HEPES, pH 7.4.

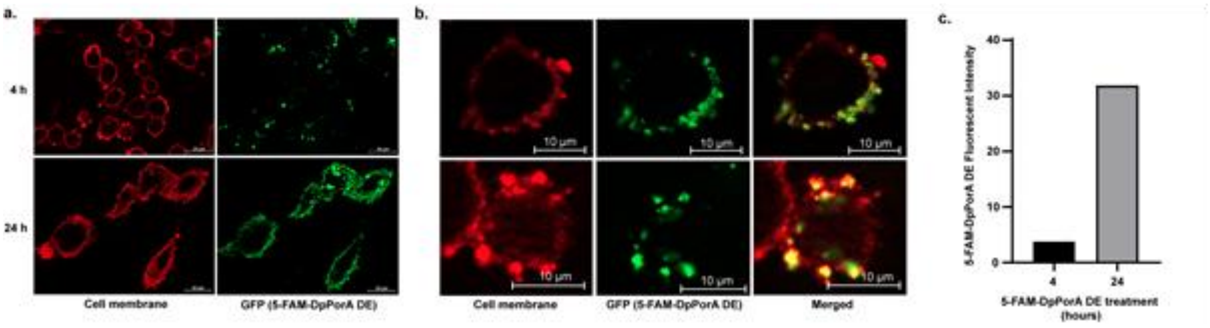


**Supplementary Fig. 22: Fluorescence study of 5-FAM-DpPorA DE.**

**a.** Representative fluorescence images showing the distribution of 5-FAM-DpPorA DE (green) in MDA-MB-231 cells at 4 hours and 24 hours post-treatment. Cell membranes were stained with a CellMask dye (red). Scale bar: 20 µm, magnification 63X, 50 cells per group. **b.** Representative high-magnification insets showing incorporation of 5-FAM-DpPorA DE into the membrane. Merged images highlight the co-localization of 5-FAM-DpPorA DE with the plasma membrane. Scale bar: 10 µm. **c.** Quantification of 5-FAM-DpPorA DE fluorescence intensity at 4 hours and 24 hours using ImageJ, representing the average fluorescent signal at different time points.

**Supplementary tables:**

| | -0.2 V | | +0.2 V | | |
|---|---|---|---|---|---|
| | $G_{MD}$ (nS) | $I_-/I_+$ | $G_{MD}$ (nS) | $I_-/I_+$ | $G_{exp}$(nS) |
| LpPorA | 2.70 ± 0.09 | 1.5 | 2.46 ± 0.50 | 1.62 | 3.0 ± 0.2 |
| DpPorA | 3.66 ± 0.96 | 1.74 | 3.31 ± 0.45 | 1.99 | 2.8 ± 0.2 |
| LpPorA DE | 2.80 ± 0.18 | 3.22 | 4.14 ± 0.23 | 2.79 | 3.8 ± 0.2 |
| DpPorA DE | 2.62 ± 0.75 | 3.94 | 3.89 ± 0.29 | 3.89 | 3.8 ± 0.2 |

| | -0.5 V | | +0.5 V | | |
|---|---|---|---|---|---|
| | $G_{MD}$ (nS) | $I_-/I_+$ | $G_{MD}$ (nS) | $I_-/I_+$ | $G_{exp}$(nS) |
| LpPorA | 3.54 ± 0.11 | 1.57 | 3.14 ± 0.36 | 1.66 | 3.0 ± 0.2 |
| DpPorA | 4.36 ± 0.18 | 1.71 | 3.94 ± 0.12 | 1.99 | 2.8 ± 0.2 |
| LpPorA DE | 3.19 ± 0.69 | 2.45 | 4.22 ± 1.04 | 2.33 | 3.8 ± 0.2 |
| DpPorA DE | 3.25 ± 0.34 | 2.99 | 4.69 ± 0.79 | 2.02 | 3.8 ± 0.2 |

**Supplementary Table 1**: **Average computed conductance values (GMD) for L and D pores**. Average conductance values $G_{MD}$ obtained from the applied-field MD simulations compared to the experimental conductance values $G_{exp}$ for the investigated pores.

| Concentration of peptide (µM) | Corresponding DDM concentration (%) |
|---|---|
| 10 | 0.0005 |
| 20 | 0.001 |
| 25 | 0.00125 |

**Supplementary Table 2: Concentrations of peptide tested and corresponding DDM concentration.**

**a.**

| Peptide | Concentration (μM) | % Cell viability (Corresponding DDM concentration) | % Cell viability (Peptide) |
|---|---|---|---|
| DpPorA | 10 | 94.82 | 99.39 |
| | 20 | 98.35 | 96.36 |
| LpPorA | 10 | 94.82 | 89.05 |
| | 20 | 98.35 | 88.65 |

**b.**

| Peptide | Concentration (μM) | % Cell viability (Corresponding DDM concentration) | % Cell viability (Peptide) |
|---|---|---|---|
| DpPorA DE | 10 | 95.4 | 89 |
| | 20 | 96.4 | 81 |
| | 25 | 99.17 | 72.79 |
| LpPorA DE | 10 | 94.3 | 88.9 |
| | 20 | 92.35 | 85.13 |
| | 25 | 99.17 | 90.74 |

**Supplementary Table 3. Effect of peptides on the viability of MDA-MB-231 cells**

**a.** Effect of DpPorA and LpPorA peptides on the viability of MDA-MB-231 cells. **b.** Effect of DpPorA DE and LpPorA DE peptides on the viability of MDA-MB-231 cells.

**a.**

| Phenotype | Treatment (Cell number %) | | |
|---|---|---|---|
| | Control | DDM | DpPorA DE |
| Intact | 67.38 | 71.3 | 0.63 |
| Disrupted | 32.62 | 28.7 | 99.37 |

**b.**

| Phenotype | Treatment (Cell number) | | |
|---|---|---|---|
| | Control | DDM | DpPorA DE |
| Intact | 95 | 82 | 1 |
| Disrupted | 46 | 33 | 158 |

**Supplementary Table 4: Intact and Disrupted phenotypes of the cell membrane.**
**a.** Percentages of Intact and Disrupted phenotypes for Control, 0.00125% DDM and 25 µM DpPorA DE treated cells. **b.** The total number of cells with Intact and Disrupted phenotypes for Control, 0.00125% DDM and DpPorA DE treated cells.

**Supplementary References:**


1. Benz, R., Schmid, A. & Hancock, R.E. Ion selectivity of gram-negative bacterial porins. *J. Bacteriol*. **162**, 722-727 (1985).
2. Krishnan R, S. et al. Assembly of transmembrane pores from mirror-image peptides. *Nat. Commun*. **13**, 5377 (2022).
3. Wood, C. W. & Woolfson, D. N. CCBuilder 2.0: Powerful and accessible coiled-coil modeling. *Protein Sci.* **27**, 103–111 (2018).
4. BIOVIA, Dassault Systèmes, Discovery Studio Visualizer, San Diego: Dassault Systèmes, (2019).
5. Evans, D. J. & Holian, B. L. The Nose–Hoover thermostat. *J. Chem. Phys*. **83**, 4069–4074 (1985).
6. Aksimentiev, A. & Schulten, K. Imaging α-Hemolysin with Molecular Dynamics: Ionic Conductance, Osmotic Permeability, and the Electrostatic Potential Map. *Biophys. J.* **88**, 3745–3761 (2005).
7. Laskowski, R. A., MacArthur, M. W., Moss, D. S. & Thornton, J. M. PROCHECK: a program to check the stereochemical quality of protein structures. *J. Appl. Crystallogr.* **26**, 283–291 (1993).
8. Towse, C.-L., Hopping, G., Vulovic, I. & Daggett, V. Nature versus design: the conformational propensities of d-amino acids and the importance of side chain chirality. *Protein Eng. Des. Sel.* **27**, 447–455 (2014).
9. Krishnan R, S. *et al.* Assembly of transmembrane pores from mirror-image peptides. *Nat. Commun.* **13**, 5377 (2022).
